# Eco-Friendly Conductive Hydrogels: Towards Green Wearable Electronics

**DOI:** 10.3390/gels11040220

**Published:** 2025-03-21

**Authors:** José María Calderón Moreno, Mariana Chelu, Monica Popa

**Affiliations:** “Ilie Murgulescu” Institute of Physical Chemistry, 202 Splaiul Independentei, 060021 Bucharest, Romania; pmonica@icf.ro

**Keywords:** eco-friendly conductive hydrogels, biomedical wearable hydrogels, sustainable materials, biodegradable conductive polymers, green synthesis, smart materials, circular economy, bio-based wearable electronics, recyclable soft materials

## Abstract

The rapid advancement of wearable electronics has catalyzed the development of flexible, lightweight, and highly conductive materials. Among these, conductive hydrogels have emerged as promising candidates due to their tissue-like properties, which can minimize the mechanical mismatch between flexible devices and biological tissues and excellent electrical conductivity, stretchability and biocompatibility. However, the environmental impact of synthetic components and production processes in conventional conductive hydrogels poses significant challenges to their sustainable application. This review explores recent advances in eco-friendly conductive hydrogels used in healthcare, focusing on their design, fabrication, and applications in green wearable electronics. Emphasis is placed on the use of natural polymers, bio-based crosslinkers, and green synthesis methods to improve sustainability while maintaining high performance. We discuss the incorporation of conductive polymers and carbon-based nanomaterials into environmentally benign matrices. Additionally, the article highlights strategies for improving the biodegradability, recyclability, and energy efficiency of these materials. By addressing current limitations and future opportunities, this review aims to provide a comprehensive understanding of environmentally friendly conductive hydrogels as a basis for the next generation of sustainable wearable technologies.

## 1. Introduction

### 1.1. Overview of Wearable Electronics (WE)

**Figure 1:**
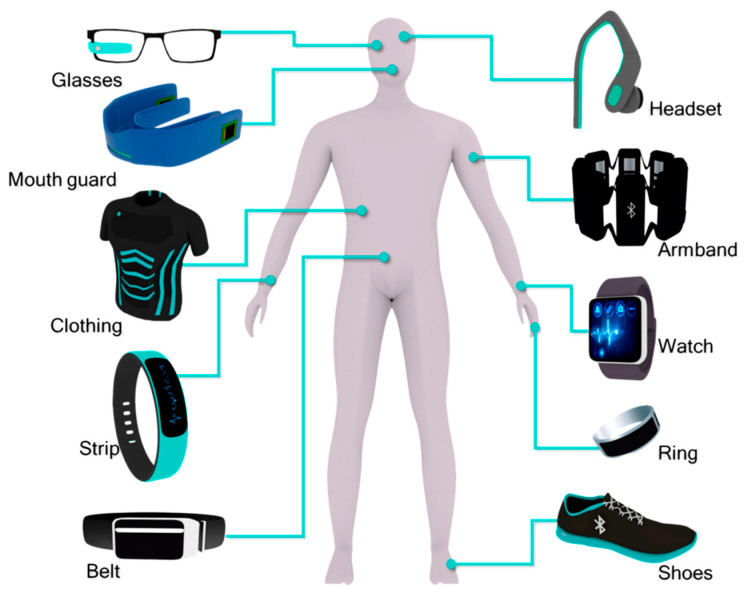
Various WE devices used in healthcare. Reprinted with permission from [5].

WE represent a rapidly evolving field that merges advanced materials, sensor technologies, and miniaturization with everyday consumer needs [1,2,3]. These devices, typically worn on the body (Figure 1), monitor, communicate, and interact with users, offering a wide range of applications from health tracking to immersive experiences [4,5]. Key areas of WE include health and fitness monitoring [6,7], medical wearables [8], augmented and virtual reality [9,10], smart clothing [11], and biometric and neural interfaces [12].

The most popular application of WE is in health and fitness monitoring devices like smartwatches and fitness trackers that collect data on heart rate, physical activity, sleep patterns, blood oxygen levels, and even stress indicators. Recent advances involve the use of biosensors for noninvasive monitoring of metabolic markers, glucose levels [13], and other biomarkers in wearable medical devices, including continuous noninvasive glucose monitors [14,15], electrocardiogram (ECG) monitors [16], contact-lens biosensors [17], and wearable insulin pumps, with the goal of improving chronic disease management. Recent developments include self-powered, skin-integrated implantable sensors [18,19] and advanced fabrics that can measure muscle activity [20], body temperature, and even detect falls or stress [1]. Research in this sector aims to create devices that provide real-time health diagnostics, drug delivery, and continuous monitoring [21], focused on enhancing the accuracy and battery life of these sensors and expanding their capabilities to monitor a broader range of health metrics that can significantly improve patient outcomes and reduce hospital visits [22,23]. Wearable devices that enable augmented and virtual reality experiences now offer improved resolution, lighter designs, and more sophisticated spatial tracking [24]. Recent research focuses on improving user immersion, minimizing motion sickness and making these devices more comfortable for extended use [25,26]. There is a growing interest in creating WE that can interact directly with the body’s biological systems [27]. Brain–computer interfaces that allow direct control of devices via neural signals are one example [28,29]. These devices can assist with everything from controlling prosthetics to providing communication solutions for people with disabilities. Current research focuses on improving signal acquisition and minimizing the invasiveness of these systems [30,31,32].

A critical challenge in WE is how to power the devices for long periods of time without compromising their functionality, size, or comfort [33]. A significant development in WE is the use of flexible, stretchable, and lightweight materials that conform to the body, such as conductive polymers, graphene, and organic electronics [34,35,36,37]. Flexible displays, integrated sensors, and energy storage devices that can bend, stretch, and conform to the skin are important components that open up new possibilities in designing more comfortable and discreet WE [38,39]. Recent innovations focus on energy-efficient systems and self-charging solutions. Energy harvesting technologies, including thermoelectric, piezoelectric, and solar power, are being integrated into wearables to extend battery life or even eliminate the need for external charging [40]. Supercapacitors, which can charge rapidly, are also being explored [41]. The evolution of wireless communication technologies, including 5G, enables seamless, low-power connectivity in WE [42]. This allows devices to communicate with each other and the cloud, facilitating real-time data processing and sharing. Artificial intelligence and machine learning are transforming wearable devices by enabling real-time data processing, predictive analytics, and personalized health recommendations [43]. For example, smartwatches can now analyze heart rate variability patterns and predict potential health risks such as arrhythmias or strokes [44]. In the future, wearables may incorporate more sophisticated algorithms for better disease prevention, early diagnosis, and mental health management [21]. Recent developments also focus on integrating multiple sensors in WE to provide comprehensive monitoring. This can include combining physiological data with environmental sensors (e.g., air quality, UV exposure), emotional state detection through facial expression recognition, or combining biometric data with machine learning to predict individual needs and behaviors [45]. As wearable devices gather more personal data, privacy and security concerns have become prominent. Ensuring that sensitive health data are protected from unauthorized access, along with maintaining user anonymity, is a critical challenge [46]. Advances in cryptography, blockchain, and decentralized data storage are being explored to safeguard wearable users’ privacy [47]. While functionality is key, another critical challenge is to achieve a sustainable customer engagement [23]. Future wearables will likely be esthetically pleasing and blend seamlessly into everyday fashion, with more collaborations between tech companies and fashion brands to blur the line between tech research and style.

Despite the rapid growth in WE, the main ongoing challenges remain: (i) battery life, as power consumption continues to be a limiting factor, especially in devices with continuous monitoring and processing requirements; (ii) comfort and design, it is a difficult task integrating high-performance sensors into small, portable devices without sacrificing comfort, esthetics, or ease of use; (iii) data management and safety, as processing and interpreting the data in a way that provides meaningful insights, as along with establishing clear regulatory pathways, is crucial to ensuring the safety and efficacy of these devices [23,27,48].

### 1.2. Importance of Conductive Hydrogels (CHG)

**Figure 2:**
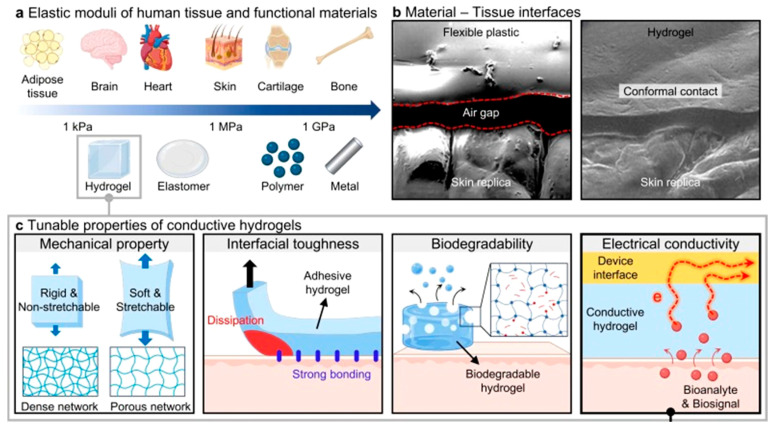
Properties of CHG used in bioelectronic devices. (**a**) Elastic moduli compared to human tissues and other conductive materials. (**b**) Gap at the interface with human skin of other materials (left) compared to conformal contact of hydrogels (right). Reprinted with permission from [56]. Copyright 2021 American Association for the Advancement of Science. (**c**) CHG tunable properties. Reprinted with permission from [12]. Copyright 2024 Elsevier.

Recently, hybridization research of conductive polymers with organogels or hydrogels has been active and is forming a new research area. CHG are an emerging class of materials gaining significant attention in the field of WE due to their unique combination of properties, such as high flexibility and stretchability, biocompatibility, and electrical conductivity [37,49,50,51]. These materials are typically composed of water-absorbing polymer networks (hydrogels) embedded with conductive components like carbon-based materials or organic conductors. The synergy between their conductive and hydrogel properties makes them ideal for a range of applications in wearable devices. Most tissues in the human body are softer than most engineering materials (Figure 2). Differences in mechanical response can lead to severe tissue damage during prolonged contact. Hydrogels have tunable elasticity and can stretch to a significant extent without losing their integrity or performance [52]. This makes them well-suited for WE applications, where flexibility and the ability to conform to the body are crucial for comfort and usability. CHG can seamlessly integrate into garments, sensors, or skin patches that adhere naturally to the skin to monitor physiological signals or provide therapeutic treatment [53,54], offering both functional and comfortable solutions for wearable devices [12,55,56].

**Figure 3:**
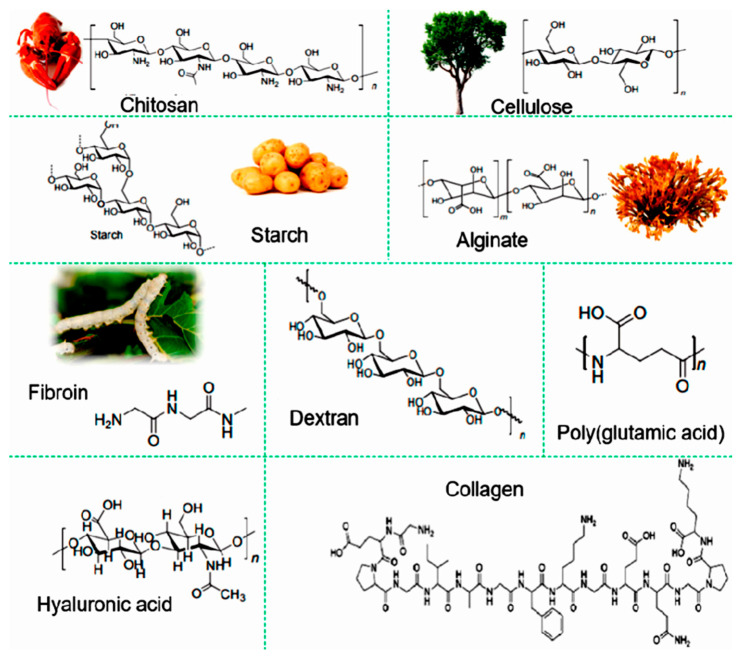
Natural biodegradable polymers. Reprinted with permission from [58]. Copyright 2021 American Chemical Society.

Many CHG are inherently biocompatible, especially those based on natural biopolymers (Figure 3). Natural biopolymers are biodegradable and affordable. They are non-toxic and safe for use on the skin or within biological systems. Their properties [57], for instance their hydrophilicity and ion permeability, make them ideal for use in many WE, particularly health monitoring devices, and even neural interfaces, where close contact with the human body or biological fluids for extended periods of time is required. Their ability to maintain a close fit with the skin also minimizes discomfort while enhancing the accuracy of measurements, such as skin temperature, hydration levels, or sweat analysis. CHG can mimic the ionic conductivity of biological tissues, allowing them to function effectively in applications that require the transmission of electrical signals, such as in sensors or electroactive devices. The presence of water within hydrogels allows for easy ion movement, making them excellent conductors of electricity in wet or moist environments. This property is particularly important in wearable biosensors and health-monitoring devices, where real-time electrical measurements (e.g., ECGs, EMGs) need to be taken with high precision.

**Figure 4:**
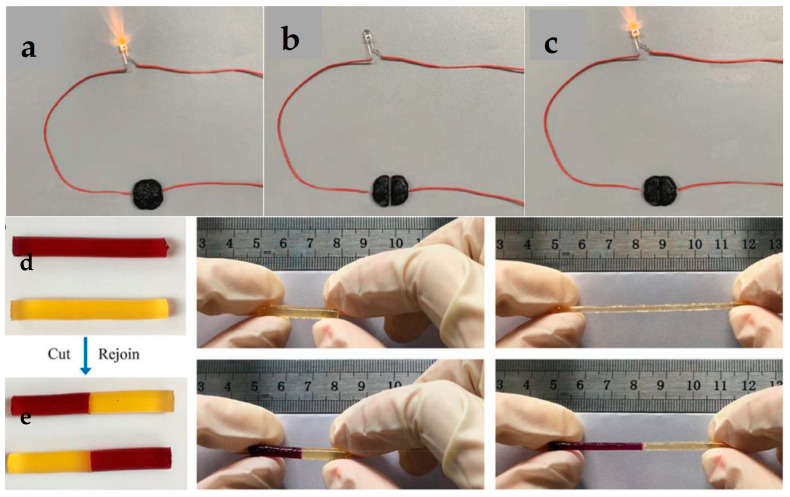
A self-healing hydrogel connected with a red LED bulb in a circuit: (**a**) original circuit, (**b**) completely broken hydrogel, and (**c**) self-healed. Reprinted with permission from [63]. Copyright 2021 American Chemical Society. (**d**) Stretching ability of a gelatin-based hydrogel and (**e**) self-healing after being cut. Reprinted with permission from [64]. Copyright 2019 American Chemical Society.

Many CHG exhibit self-healing capabilities (Figure 4), meaning that after being damaged (such as from stretching or puncturing), they can recover their mechanical and electrical properties without external intervention [59,60]. For instance, if a hydrogel-based sensor embedded in a wearable device is strained or cut, it can naturally heal and continue to provide accurate data. These self-healing capabilities extend the lifespan of wearable devices by ensuring continued functionality despite wear and tear, a particularly useful attribute in WE, where mechanical stress is common.

Hydrogels show different responses to changes in environmental stimuli, such as changes in temperature, humidity, or pH [52]. This property is crucial for applications like sweat sensors or skin moisture monitoring, where accurate detection of environmental changes is needed. For example, in sportswear or medical wearables, hydrogels can monitor hydration levels, detect biomarkers in sweat, or track temperature variations, providing valuable real-time data to the wearer. The ability of CHG to retain water and exhibit ionic conductivity can be employed in energy storage and harvesting applications and makes them potential candidates for use in supercapacitors and batteries, where efficient energy storage is needed in compact, flexible, and lightweight formats [61]. As WE increasingly require integrated power solutions, CHG can provide energy storage that conforms to the human body without the bulkiness or rigidity of traditional batteries [62].

Despite their promising properties, several challenges remain in the development and widespread adoption of CHG in WE: (I) Stability, CHG can be susceptible to dehydration over time, which can affect their performance; it is necessary to improve the long-term stability and durability of CHG under various environmental conditions. (II) Scalability, large-scale production of CHG while maintaining consistent quality and performance remains a challenge; therefore, advanced manufacturing techniques need to be developed to produce these materials efficiently and cost-effectively for mass-market applications. (III) Integration with other materials: while CHG offer excellent performance on their own, integrating them seamlessly with other materials used in WE (such as sensors, circuits, and batteries) poses challenges. Innovations in hybrid materials that combine hydrogels with other conductors or energy storage materials are necessary for the development of fully functional wearable devices.

Specific solutions to address each challenge are:

(I) Stability against dehydration: dehydration reduces hydrogel conductivity and flexibility, affecting long-term performance. Solutions include water retention strategies: incorporating hydrophilic polymers and humectants, such as glycerol, polyethylene glycol, or sorbitol, to retain moisture by forming hydrogen bonds with water molecules or ionic liquids (e.g., choline chloride-based) that prevent water evaporation while maintaining ionic conductivity; crosslinking strategies for water binding, as double-network hydrogels that combine covalent and physical crosslinking (e.g., polyacrylamide-alginate) to trap water within a stable matrix, e.g., polyacrylamide-clay hydrogels prevent water loss while maintaining flexibility [65]; introduction of hydrophobic domains (e.g., fluorinated or silicone-based groups) to reduce evaporation; surface coatings or encapsulation by protective layers (e.g., mussel-inspired polydopamine or zwitterionic coatings) to minimize water loss; elastomer encapsulation by thin layers of silicone allow flexibility while limiting dehydration [66].
(II) Scalability for mass production: large-scale production must be cost-effective, energy-efficient, and repeatable to meet mass-market demands. Use of natural biopolymers, cellulose, chitosan, gelatin, and alginate-based conductive hydrogels reduce costs compared to synthetic polymers, as well as reduced reliance on costly carbon nanomaterials (CNTs, graphene) by optimizing MXenes, PEDOT:PSS, or metal nanowires. PEDOT:PSS with crosslinked biopolymers provides affordable conductivity. Use of high-throughput fabrication methods, such as 3D printing and direct ink writing; extrusion-based printing of hydrogel networks ensures precision while reducing material waste. Scalable crosslinking and polymerization by UV rapid photopolymerization and sustainable methods using enzyme-catalyzed gelation (e.g., horseradish peroxidase for silk-based hydrogels) reduce reaction time compared to traditional thermal curing [67].
(III) Integration with other materials: wearable electronics require hydrogels to integrate with metals, polymers, and textiles while maintaining flexibility and conductivity. Hydrogel–elastomer blends combine hydrogels with silicone or polyurethane elastomers to improve mechanical strength [68]. Electrospun nanofiber reinforcement of hydrogels improves mechanical stability. MXenes, silver nanowires, CNTs, and graphene enhance conductivity while maintaining stretchability. Ionic liquids (e.g., LiCl, NaCl solutions) for ionically conductive hydrogels or carbon-based fillers for electronic conductivity enhance the conductivity. For better skin adhesion, mussel-inspired polydopamine coatings enhance adhesion between hydrogel and stretchable electronics [69]. Interpenetrating polymer networks enhance compatibility with soft substrates like textiles and silicone.

By addressing stability, scalability, and integration, conductive hydrogels can transition from lab research to real-world WE. Combining moisture-retention strategies, scalable manufacturing, and hybrid material integration will be key to unlocking commercial applications in smart textiles and biosensors.

## 2. Material Components of Eco-Friendly CHG

### 2.1. Natural Polymers

For CHG to be truly sustainable and eco-friendly, their base components need to be sourced from natural, renewable, and biodegradable materials (Figure 3). This section explores the material components of eco-friendly CHG, with a particular focus on natural polymers [63] and their integration into WE. The integration of natural polymers into eco-friendly CHG offers several advantages for WE. These biopolymers, derived from renewable sources like plants, algae, animals, or microorganisms, are biodegradable, making them suitable for sustainable applications in WE, and biocompatible, making them ideal for applications that involve direct contact with the skin, such as biosensors, health monitors, and flexible electronics [70,71]. Natural polymers, such as alginate, chitosan, cellulose, and gelatin, are ideal candidates for being the structural backbone of eco-friendly hydrogels [72,73,74]. When combined with conductive additives like graphene or conducting polymers, these natural polymer-based hydrogels can provide high electrical conductivity, mechanical flexibility, and moisture retention, making them well-suited for wearable applications [75]. Many natural polymers and conductive additives are lightweight and flexible, reducing the energy requirements for their production and operation in wearable devices [76]. Using renewable, biodegradable, and eco-friendly materials helps reduce the environmental impact of WE, addressing concerns related to e-waste and resource depletion [77].

Common natural polymers used in eco-friendly CHG include carbohydrate-based polysaccharides, protein polymers, and polyphenolic compounds. Polysaccharides are one of the most widely used groups of natural polymers in the development of hydrogels due to their excellent water retention capacity, biocompatibility, and biodegradability [78]. Cellulose, found in plant cell walls, is one of the most abundant natural polymers on Earth. It is biodegradable and renewable, making it an ideal candidate for eco-friendly hydrogels. Cellulose derivatives, such as carboxymethyl cellulose, are commonly used to enhance the structural integrity and water retention of hydrogels. By incorporating conductive nanoparticles, polymers, or graphene, cellulose-based hydrogels can also be made electrically conductive. These conductive cellulose-based hybrid materials exhibit excellent physicochemical properties, biodegradability, and biocompatibility [34,79,80,81,82]. Starch, like cellulose, is a natural polymer formed by glucose monomers and is used for flexible electronic sensors. Progress regarding starch-based WE has been recently reviewed [83]. Derived from brown seaweed, alginate is a versatile biopolymer used in hydrogels for biomedical and electronic applications [84]. Alginate is extracted from seaweed and can be crosslinked using divalent cations like calcium ions, making it an eco-friendly choice for hydrogel fabrication. The process does not require toxic solvents or chemicals, and alginate is biodegradable, making it an ideal material for environmentally friendly CHG [67]. Alginate hydrogels are highly hydrophilic, forming a gel-like structure that retains water effectively. Alginate can be chemically modified to enhance its mechanical properties and conductivity when combined with conductive materials like graphene or conductive polymers [85].

Chitosan is a natural biopolymer obtained from the shells of crustaceans such as shrimp and crabs that can form hydrogels through physical or chemical crosslinking [86]. It is biodegradable, biocompatible, and can be synthesized using water-based methods, eliminating the need for harmful solvents, and can be modified to improve its mechanical and conductive properties. Chitosan-based hydrogels are often used in biomedical applications and can serve as a matrix for embedding conductive materials to create electroactive hydrogels for wearables [87]. Pectin, a carbohydrate polymer found in fruits, particularly citrus fruits, is another polysaccharide used in the development of hydrogels [88]. It has excellent gelation properties and can be chemically modified to increase its water-holding capacity and conductivity. Pectin-based hydrogels offer a sustainable alternative for WE applications [89].

Proteins are natural polymers derived from animal and plant sources and are characterized by their biocompatibility and biodegradability. Protein-based hydrogels are becoming increasingly popular for eco-friendly WE due to their flexibility and ability to form conductive networks. Silk fibroin, derived from the silkworm, is a protein that has gained attention for its mechanical strength, flexibility, and biocompatibility. It is used in the development of hydrogels for wearable devices and biosensors [90,91]. Silk fibroin can be combined with conductive materials like carbon nanotubes (CNTs) or graphene to create CHG that are both environmentally friendly and highly functional. Gelatin, a protein obtained from animal collagen, is another natural polymer commonly used in hydrogel applications [64]. Gelatin-based hydrogels are biodegradable and can be engineered to improve their mechanical properties and water retention. Figure 4 illustrates the stretching (Figure 4d) and self-healing (Figure 4e) abilities of a gelatin-based hydrogel, based on the Schiff base reaction due to the imine bonds formed between amino-gelatin and carboxymethyl cellulose. Gelatin was reacted with ethylenediamine to increase the content of amino groups, and then dialdehyde carboxymethyl cellulose was used to cross-link amino-gelatin to fabricate the self-healing hydrogel [64].

Polyphenolic compounds, such as tannins and lignin, are natural polymers that can be extracted from plants and that offer both mechanical and electrical conductivity when properly processed. Lignin, a complex polymer found in plant cell walls, has recently been explored as a sustainable material for hydrogels. Lignin is abundant and biodegradable, and it has intrinsic conductivity, making it a promising component for eco-friendly CHG. Lignin-based hydrogels can be used in wearable sensors, providing an environmentally sustainable alternative to synthetic conductive materials. Tannins are polyphenolic compounds found in a variety of plants. They are biodegradable and have been shown to form conductive networks when incorporated into hydrogels. Tannin-based hydrogels are an emerging area of research, particularly for applications in sensors and energy storage devices.

### 2.2. Conductive Materials for Natural Polymer Hydrogels

**Figure 5:**
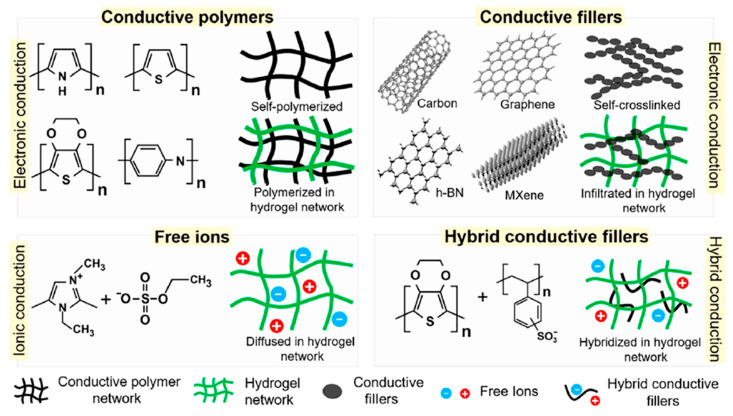
Structures of different CHG networks. Reprinted with permission from [93]. Copyright 2020 American Chemical Society.

While natural polymers provide the structural matrix for hydrogels, the addition of conductive materials is necessary to make them electrically active [92,93], consequently forming a smart hydrogel, endowed with electrical conductivity, electrochemical, and electromechanical properties [94]. Figure 5 illustrates different types of CHG structures: self-polymerized conductive polymers; hydrogel networks interpenetrated by conductive fillers; structures containing free ions; and hybrid structures containing a mixture of them, embedding conductive fillers/polymers/free ions into an existing non-conductive hydrogel matrix. Highly CHG can be fabricated by the addition of liquid metals or silver nanowires [7], but these conductive materials need to be eco-friendly and compatible with the natural polymers used in the hydrogel. Some commonly used conductive additives in eco-friendly conductive CHG include conductive polymers, organic materials that can conduct electricity [95]. They are a key component in enhancing the conductivity of natural polymer-based hydrogels. Polypyrrole (PPy) is a widely used conducting polymer that can be incorporated into natural polymer hydrogels. It can be synthesized from renewable resources and is biodegradable, making it an eco-friendly option for WE [96,97]. PPy has good stability and conductivity, making it suitable for use in sensors and other electronics. Polyaniline (PANI) is another conductive polymer that is often used in the fabrication of CHG. PANI is synthesized from aniline and can be doped to enhance its conductivity. It is stable, cost-effective, and can be combined with natural polymers like alginate or chitosan to create CHG for WE [98]. Poly(3,4-ethylene dioxythiophene):poly(styrene sulfonate) (PEDOT:PSS) is a composite material in which PEDOT (the conductive polymer) provides electrical conductivity, and PSS acts as a counter-ion to balance the charge and improve the water solubility and processability. These conductive polymers, PPy, PANI, and PEDOT, exhibit electrical conductivity due to the presence of delocalized π orbitals, allowing electron movement along the polymeric chains. They are some of the most widely used conducting polymers because of their ease of preparation, tunable electrochemical properties, and environmental friendliness [99,100]. Carbon-based materials, such as graphene, CNTs, and activated carbon, are frequently used to improve the conductivity of natural polymer hydrogels [101]. These materials have high surface areas and excellent electrical conductivity. Graphene, a one-atom-thick sheet of carbon atoms arranged in a hexagonal lattice, is a highly conductive material that can be easily integrated into natural polymer hydrogels. Graphene-based CHG are lightweight, flexible, and can be used in wearable devices for health monitoring, energy storage, and sensors. CNTs are cylindrical structures made of carbon atoms with excellent electrical and mechanical properties. CNTs are often embedded into hydrogels to enhance their conductivity while maintaining the flexibility and stretchability required for wearable applications. They can be combined with polysaccharides like alginate or chitosan to create eco-friendly CHG with increased durability and enhanced electrophysiological activity [102,103,104]. Derived from the pyrolysis of organic materials, biochar is a renewable, sustainable material with electrical conductivity. Biochar-based CHG are emerging as an eco-friendly alternative to synthetic conductive agents, offering a way to recycle organic waste materials while enhancing the conductivity of hydrogels [105]. Although ionic charge carriers tend to destroy the hydrogen-bonding network, natural polymers can be endowed with ionic conductivity by the introduction of ionic liquids [106] or free ions, for instance by salt solution soaking [107,108].

Research is increasingly focused on developing bio-inspired conductive materials that mimic the properties of biological tissues [82]. These materials, often derived from natural sources, are designed to be more environmentally friendly and capable of integrating into living systems [74].

### 2.3. Green Crosslinkers and Additives

The choice of green crosslinkers and additives is essential in the fabrication of eco-friendly CHG for WE applications. Green additives enhance the physicochemical and mechanical properties, allowing them to be flexible, stretchable, and durable, which is essential for wearable devices, while ensuring that the CHG remain biocompatible and biodegradable, safe for skin contact, and with reduced environmental impact. For instance, unmodified chitosan has very limited applications due to its poor solubility at neutral pH, low porosity, and poor mechanical properties. Consequently, crosslinking becomes necessary. Crosslinking is the process by which polymer chains are chemically bonded to each other, leading to the formation of a network structure that imparts mechanical strength, stability, and the ability to retain water in hydrogels [52,109]. In eco-friendly CHG, green crosslinkers derived from renewable, non-toxic, and biodegradable sources, contribute to the sustainability and environmental integrity. These crosslinkers must enable the formation of hydrogels with excellent mechanical properties while being biodegradable and non-toxic. Genipin is a naturally occurring water-soluble crosslinker derived from the gardenia fruit [110], widely used in biodegradable materials due to its low toxicity and ability to form crosslinked networks in biopolymers like chitosan, alginate, and gelatin. Genipin crosslinks hydrogels through the formation of covalent bonds between hydroxyl and amine groups, improving the stability and mechanical strength of the hydrogel without compromising its biocompatibility [111]. Modern crosslinkers, however, tend to use physical forces such as electrostatic interactions, H-bonding, and hydrophobic interactions to establish crosslinks across the chitosan chains [112,113]. Natural biopolymer-based polyelectrolyte complexes (PEC), formed by electrostatic interactions between two oppositely charged biopolymers, have been attracting increasing attention in recent years for their potential in tailoring specific applications [114,115]. Tannic acid, a polyphenolic compound found in plants, is a green crosslinker that can form stable crosslinks with proteins, polysaccharides, and other biopolymers [116]. It enhances the mechanical properties and water retention capacity of hydrogels. Tannic acid has been used to crosslink chitosan-based hydrogels, making them more durable for use in WE. Under UV light, riboflavin (vitamin B2), a naturally occurring vitamin, has been employed as a crosslinker in hydrogels [117,118]. This approach is environmentally friendly, as riboflavin is both renewable and biocompatible, making it ideal for creating sustainable and functional hydrogels for WE. Citric acid, a naturally occurring organic acid found in citrus fruits, has been explored as an eco-friendly crosslinker for biopolymers [119]. It forms ester bonds with hydroxyl groups in polysaccharides like cellulose, making it an eco-friendly alternative for creating stable, flexible, and biodegradable hydrogels. Epoxy resins derived from plant oils such as soybean or linseed oil can be used as green crosslinkers in the fabrication of hydrogels [120]. These resins undergo chemical reactions that create a crosslinked network of polymers, providing the desired mechanical strength and durability while being biodegradable. Green crosslinkers derived from natural sources such as genipin, tannic acid, and citric acid contribute to the overall biodegradability of the hydrogel, ensuring that the material breaks down naturally over time without harming the environment. Unlike many synthetic crosslinkers, green crosslinkers derived from renewable natural resources are often biocompatible, which is essential for WE that come into contact with skin and biological tissues and contribute to a more sustainable production process.

In addition to crosslinkers, various eco-friendly additives derived from natural sources are used to enhance the mechanical properties and provide specific functionalities of CHG needed in WE, such as plasticizers or softening agents, natural stabilizers, antioxidants, and natural dyes. Glycerol, a naturally derived plasticizer, is commonly used to improve the flexibility and water retention of hydrogels [121]. By reducing the stiffness of the polymer network, glycerol helps to maintain the mechanical integrity of the hydrogel, ensuring that it remains flexible and functional for wearable applications. Sucrose, a naturally occurring sugar, can also be used as a plasticizer to enhance the flexibility and water content of hydrogels [122]. It contributes to the formation of a more pliable and stretchable material, which is crucial for wearables that need to conform to the skin or body. Vitamin E (tocopherol) is a natural antioxidant that can be incorporated into CHG to improve their stability and prevent oxidative degradation. It can also enhance the longevity of the wearable device by protecting the hydrogel from environmental factors such as exposure to UV light or atmospheric oxygen [123]. Polyphenols, especially from green tea, are potent antioxidants that can stabilize the hydrogel matrix and protect it from degradation over time [124]. These polyphenols also contribute to the eco-friendly nature of the hydrogel, as they are derived from natural sources and have minimal environmental impact. Anthocyanins are water-soluble pigments found in fruits and vegetables that can be used to color hydrogels [125]. In addition to providing esthetic value, anthocyanins have antioxidant properties that can help protect the hydrogel from oxidative damage, making them useful for WE that need to remain durable over time. Derived from turmeric, curcumin is a natural dye with anti-inflammatory and antioxidant properties. It has been used as a green additive in hydrogels to improve their functional properties, particularly in the context of bioactive and medical wearable devices [126].

## 3. Green Synthesis Approaches

### 3.1. Solvent-Free and Aqueous Synthesis Methods

Green synthesis approaches are emerging as an essential strategy to produce eco-friendly materials for various applications, including WE. In the context of CHG, green synthesis aims to create materials using renewable resources, minimizing environmental impact, reducing energy consumption, and avoiding toxic chemicals. This approach aligns with the principles of green chemistry, which focuses on sustainable, non-toxic, and energy-efficient methods. This section discusses key green synthesis techniques used to produce CHG for WE, highlighting the role of renewable raw materials and environmentally friendly processes.

The synthesis of the hydrogel matrix itself can be performed using renewable, non-toxic, and biodegradable materials while reducing energy consumption and harmful byproducts [127,128,129]. The synthesis of conductive polymers using green approaches is critical to the sustainability of WE. Traditionally synthesized using hazardous chemicals and solvents, green synthesis methods for PPy focus on using natural, non-toxic solvents such as water or alcohols and renewable monomers [130]. Bio-derived pyrrole monomers, or pyrrole derivatives extracted from natural sources, can be polymerized using environmentally benign methods, such as electrochemical or enzyme oxidative polymerization [131,132,133], using mild conditions. Enzyme catalysis helps synthesize a more defect-free polymer as the polymerization occurs in a more controlled manner at low temperatures. Green synthetic routes of PANI, another widely used conductive polymer, focus on using natural oxidants like hydrogen peroxide or plant-based extracts, such as tannins or polyphenols, to facilitate polymerization [134]. These green oxidants replace toxic chemical agents like ammonium persulfate and improve the overall sustainability of the synthesis process. Other bio-based polymers, such as conducting polyphenols [135] or lignin derivatives [136], are often derived from natural sources like wood or plants and can be polymerized using water-based or low-energy methods. These green alternatives reduce the reliance on synthetic and petroleum-based materials, providing an eco-friendly path to conductive polymers.

The use of green solvents, such as water, ethanol, or ionic liquids, is an important aspect of green synthesis approaches that reduces the environmental footprint of the synthesis process and improves the material’s sustainability. These solvents are safer, non-toxic, and often biodegradable, replacing harmful solvents like chloroform, dichloromethane, or benzene. Electrochemical polymerization is an environmentally friendly method to synthesize conductive polymers like PPy and PANI [98,130]. This method uses electric current to drive the polymerization reaction, eliminating the need for toxic chemical oxidants or solvents. Electrochemical polymerization can be performed in aqueous solutions, making it a sustainable alternative to traditional chemical polymerization processes. Furthermore, this technique allows for precise control over the polymer’s properties, such as thickness and conductivity, making it well-suited for use in WE [76,137].

Graphene and CNTs are often incorporated into CHG to enhance their electrical properties. Traditional synthesis methods typically involve hazardous gases and high-energy processes [138,139]. Green synthesis approaches involving the use of renewable materials and safer processes, such as biomass or plant-based precursors, are increasingly being explored to produce these nanomaterials in a more environmentally friendly manner. For example, cellulose or lignin-based biomass can be converted into CNTs through pyrolysis or hydrothermal processes [140]. These methods eliminate the need for fossil fuels and reduce the environmental impact of CNT production. Graphene oxide (GO) is a precursor to graphene and is commonly used in the preparation of CHG. The reduction in GO to reduced graphene oxide (rGO) is typically performed using harsh reducing agents such as hydrazine, which is toxic and hazardous [141]. Green reduction methods replace these toxic agents with natural reducing agents, such as plant extracts (e.g., tea, coffee, or fruit extracts) or ascorbic acid (vitamin C). These green reducing agents are often rich in antioxidants, which can effectively reduce GO without harming the environment. Plant-based extracts contain polyphenolic compounds and flavonoids that have reducing properties. For example, extracts from green tea, grape seeds, or pomegranate are used to reduce GO to rGO in aqueous solutions [142]. This method not only eliminates toxic chemicals but also adds natural antioxidants to the system, improving the hydrogel’s stability and functionality [143,144].

### 3.2. Biocompatible Crosslinking Techniques

Crosslinking is necessary for enhancing the mechanical properties and stability of hydrogels [145]. Traditional crosslinking methods often rely on toxic reagents like glutaraldehyde [146]. However, green crosslinking methods are being developed to replace these toxic agents with safer, more sustainable alternatives (Table 1). As discussed earlier, crosslinkers like genipin (derived from the gardenia fruit), tannic acid (from plant polyphenols), and citric acid (from citrus fruits) are gaining popularity as environmentally friendly alternatives to synthetic crosslinkers. These natural crosslinkers form stable networks in hydrogels without relying on toxic chemicals. Ionic crosslinking is a common and biocompatible method used to crosslink polysaccharides, especially alginate, by introducing divalent cations like calcium ions (Ca^2+^) [147,148]. The calcium ions bind to the carboxyl groups in the alginate, forming a gel network. This process does not require any toxic chemicals and is performed in an aqueous environment, making it highly suitable for biomedical and wearable applications.

Physical crosslinking techniques do not require the use of chemicals, instead relying on physical forces or environmental conditions such as temperature, pH, or UV light. These methods are particularly useful for creating biocompatible hydrogels because they eliminate the need for chemical crosslinkers, reducing the risk of irritation or toxicity [149]. Thermal crosslinking utilizes heat to induce the crosslinking of polymers, such as polyvinyl alcohol (PVA), agarose, or gelatin, without the use of any toxic chemicals. This process involves heating the polymer solution to a temperature that allows the formation of crosslinks between polymer chains, resulting in the formation of a hydrogel network [12]. In some cases, the temperature is used to promote physical changes in the material, such as gelation or phase transitions, which help to stabilize the structure. Photopolymerization is a green crosslinking method that uses light (typically UV light) to initiate the formation of covalent bonds between polymer chains in a hydrogel matrix and induce polymerization, eliminating the need for chemical crosslinkers [150]. Compared with other chemical crosslinking, photocrosslinked hydrogels can achieve fast in situ polymerization crosslinking in mild reaction conditions, and low reaction heat release. The main benefit of UV crosslinking is its precise control over the crosslinking process, allowing for easy creation of complex shapes and structures [151]. This method is often used with natural polymers that contain unsaturated bonds and can be used to create hydrogels with controlled properties, such as hydrogels with adjustable mechanical strength and conductivity, without producing hazardous byproducts. Freeze–thaw crosslinking is a physical method where hydrogels are subjected to alternating freezing and thawing cycles. This technique relies on the formation of ice crystals during freezing, which helps in the physical crosslinking of polymers, such as PVA, agarose, or chitosan. The repeated freeze–thaw cycles result in a highly crosslinked hydrogel network [152].

**Table 1:** Summary of ecological crosslinking methods.

Method	Material/ Agent Used	Advantages	Disadvantages	Reference
Chemical crosslinking	Genipin Tannic acid Citric acid Caffeic acid	Non-toxicity, sustainability, bioavailability.	Requires high concentrations of agents. Insufficient mechanical strength.	[146]
Ionic crosslinking	Ca^2+^, Cu^2+^, Fe^3+^, Ag^+^	Forms stable 3D interconnected hydrogel structures and extremely robust. High mechanical strength and stability, antibacterial properties drug delivery purposes.	CaCl_2_ leads to rapid and difficult to control gelation. CaSO_4_ and CaCO_3_ reduce the gelation rate and increase the working time. Limited long-term stability under physiological conditions.	[147]
Thermal crosslinking	Polyvinyl alcohol, agarose, gelatin	Formation of the 3D network in a single step, in situ. Thermal and chemical stabilities of network structure.	Weak viscoelasticity. Easily degradable Poor mechanical properties.	[153]
Photo- polymerization	UV light	Rapid in situ crosslinking. Elimination of toxic agents.	Intricate alteration procedure. Storage conditions impact variability. Prone to bacterial contamination.	[150,151]
Freeze–thaw crosslinking	Polysaccharides, polyvinyl alcohol	No organic solvents and toxically crosslinking agents. Tunable structural, mechanical, biological properties.	Challenging uniform mixing of the initial polymer solution. Deterioration of mechanical properties. Uneven pore formation. Weak spots, fractures, or cracks.	[152]
Enzymatic crosslinking	Transglutaminase, Laccase, Peroxidase, Tyrosinase	Decreases food allergenicity by eliminating organic solvents and toxic crosslinking agents.	Limited broad-spectrum substrate.	[154,155,156]

Enzymatic crosslinking is a biocompatible approach where natural enzymes are used to catalyze the crosslinking of polymer chains [154]. This method is especially useful for hydrogels based on biopolymers like collagen, gelatin, or alginate, as enzymes can specifically target functional groups (e.g., amines, hydroxyls) in these materials, forming covalent crosslinks without the need for synthetic chemical agents. Transglutaminase is an enzyme that catalyzes the formation of covalent bonds between amine groups and glutamine residues in proteins, making it a useful crosslinking agent for protein-based hydrogels [155,156]. It has been applied in the fabrication of gelatin and collagen hydrogels that can be used in medical devices and WE. Laccase and peroxidase are enzymes that catalyze the crosslinking of phenolic compounds in natural polymers. These enzymes are often used to crosslink polysaccharides or proteins in a highly selective and controlled manner [157]. The use of these enzymes for crosslinking hydrogels avoids the need for chemical crosslinkers and ensures that the final product is biocompatible.

Green synthesis approaches for the production of eco-friendly CHG are transforming the way materials are made for WE. By focusing on renewable, non-toxic, and biodegradable materials, green chemistry principles help create sustainable solutions that not only improve the performance of wearables but also reduce their environmental impact. From bio-based conductive polymers and nanomaterials to environmentally friendly crosslinking methods, green synthesis is paving the way for the next generation of eco-friendly wearable devices. As the demand for sustainable technologies continues to grow, green approaches that require lower temperatures and less energy than traditional methods will play an essential role in shaping a more sustainable future for WE, making them more energy-efficient and cost-effective for large-scale production.

## 4. Functional Properties of Eco-Friendly CHG for WE

Eco-friendly CHG are gaining significant attention in WE due to their unique combination of flexibility, biocompatibility, and environmental sustainability. These hydrogels are designed to exhibit not only high conductivity but also favorable mechanical, thermal, and chemical properties, making them ideal for use in applications such as bioelectronics, sensors, energy storage, and health-monitoring devices [158]. This section outlines the key functional properties of eco-friendly CHG that make them suitable for WE.

### 4.1. Electrical Conductivity

One of the most critical properties for CHG in WE is their electrical conductivity. The incorporation of conductive polymers (e.g., PPy, PANI) or conductive fillers, such as carbon-based materials (e.g., graphene, CNTs) into natural polymer matrices enables to create hydrogels that exhibit high conductivity [63,159,160,161,162] and that can be used in bioelectronic sensors, energy storage devices (e.g., supercapacitors) [163], and flexible electrodes for wearable health-monitoring systems.

Eco-friendly CHG possess unique electrical properties that arise from their structure, combining a hydrated polymer network with conductive materials: on the one hand, efficient ion transport due to their water-rich structure, making them excellent electrolytes for energy storage devices and bioelectronic sensors [62]. Typical ionic conductivity ranges from 10⁻^6^ to 1 S/m, depending on composition and hydration levels [164]. Conductivities higher than 3 S/m have been reported by adding hydroxypropyl cellulose biopolymer fibers, followed by soaking in a salt solution inside a PVA hydrogel matrix [165] and in PVA/sodium alginate hydrogels through salt solution soaking [166]. On the other hand, hydrogels are generally poor electronic conductors due to the insulating nature of hydrophilic polymer chains [95]. However, by incorporating conductive materials, hydrogels can gain electronic conductivity [95,103]. The electrical properties can be adjusted by varying factors like hydration level, crosslinking density, and dopant concentration, for precise control of electrical behavior in applications such as sensors and energy storage. Unlike rigid conductors, CHG can maintain conductivity even under large deformations (stretching, bending, twisting) and after many stretching cycles, making them ideal for wearable applications. Wang et al. reported stretch-induced conductivity enhancement (6000x) in highly conductive CHG [164]. Self-healing hydrogels, in which broken conductive pathways reconnect after mechanical damage, restoring electrical functionality, are useful for durable and long-lasting wearable bioelectronics [63].

**Figure 6:**
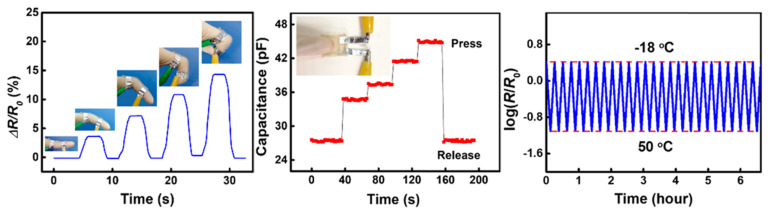
Performance of cellulose hydrogel sensors: (**left**) piezoresistive strain sensor, (**center**) capacitive pressure sensor, and (**right**) piezoresistive temperature sensor. Reprinted with permission from [162]. Copyright 2019 American Chemical Society.

Many CHG exhibit excellent electrochemical performance, with high capacitance and charge storage ability, making them suitable for supercapacitors and batteries. Hydrogels have adjustable dielectric constants, enabling their use in capacitive sensors for motion detection, touch interfaces, and WE. Unlike many traditional conductors, CHG mimic the electrical properties of natural tissues, making them ideal for bioelectronic interfaces like neural electrodes, ECG, and EEG sensors. Figure 6 shows the responses of cellulose-based hydrogel sensors [162] exhibiting stable sensitivity to tensile strain, compressive pressure, and temperature over a wide range, including subzero temperatures. CHG-based sensors can detect physiological signals (e.g., heart rate, temperature, or sweat analysis) and enable real-time data transmission.

Tunable ionic conductivity is a key feature of CHG, particularly for applications involving electrochemical energy storage, sensors, and actuators. Ionic conductors in the hydrogel facilitate the movement of ions within the network, leading to effective charge storage and transport. This property is especially important for flexible and WE that require low-voltage, energy-efficient operation, particularly in the area of flexible triboelectric nanogenerators (TENGs) [167]. CHG with a high ionic conductivity are explored for use in energy storage devices such as supercapacitors or flexible batteries that can be integrated into smart clothing or patches, to act as a charge storage material, providing a sustainable power source for electronic devices while maintaining the benefits of flexibility and biocompatibility [168].

### 4.2. Mechanical Properties and Stretchability

The mechanical properties of hydrogels for WE must be tailored to ensure they can withstand the dynamic movements of the human body. Green hydrogels generally have lower mechanical strength compared to synthetic polymer-based hydrogels, due to weaker intermolecular interactions and lower crosslinking density. They tend to be softer and more brittle, and usually have lower elasticity and stretchability, making them less suitable for load-bearing applications [52]. Synthetic hydrogels tend to have higher mechanical strength and durability due to tunable crosslinking, allowing for stronger covalent or ionic bonding, and can be engineered for high flexibility and stretchability, making them a preferred option for applications requiring repeated deformation (e.g., tissue engineering, flexible electronics). The mechanical properties of green hydrogels vary significantly based on their composition, crosslinking method, and environmental conditions. Below are typical ranges for key mechanical properties:

Compressive strength (10–500 kPa): alginate: ~50–200 kPa, chitosan: ~100–300 kPa, gelatin: ~10–100 kPa, compared to 0.1–10 MPa for synthetic hydrogels: polyacrylamide: ~100 kPa–1 MPa, polyethylene glycol: ~500 kPa–5 MPa; elastic modulus (0.1–10 MPa): cellulose-based: ~1–10 MPa, alginate: ~0.1–1 MPa, gelatin: ~0.1–0.5 MPa, compared to 1 MPa–1 GPa for synthetic hydrogels; tensile strength (10 kPa–1 MPa): alginate: ~50–500 kPa, chitosan: ~100–800 kPa, gelatin: ~10–300 kPa, compared to 0.1–5 MPa for synthetic hydrogels: polyethylene glycol: ~0.5–2 MPa, polyacrylamide: ~1–5 MPa; fracture energy or toughness (10–1000 J/m^2^): gelatin ~10–200 J/m^2^, chitosan ~100–500 J/m^2^, alginate ~50–800 J/m^2^), compared to 100–10,000 J/m^2^ for synthetic hydrogels.

**Figure 7:**
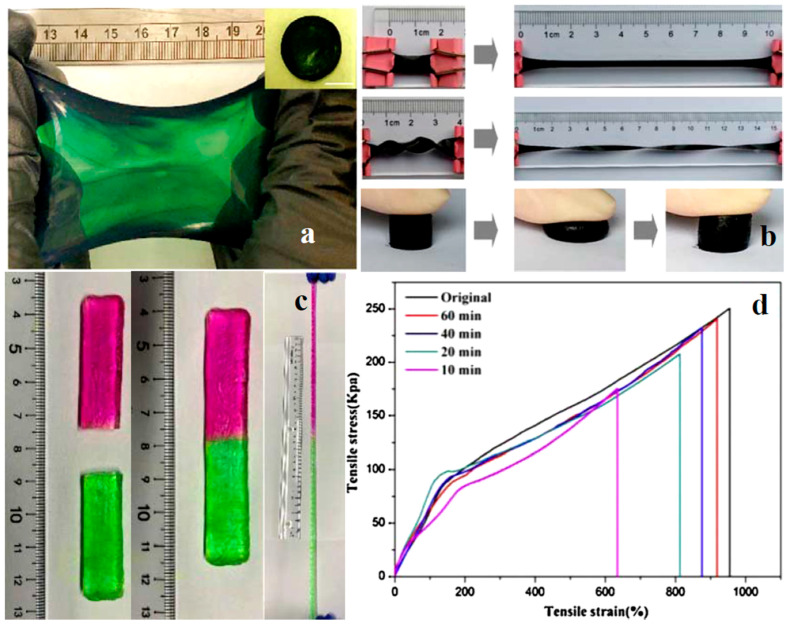
(**a**) Excellent biaxial stretchability of a green hydrogel. Reprinted with permission from [173]. Copyright 2021 Elsevier. The inset shows the hydrogel sample before stretching (scale bar, 1 cm). (**b**) Elasticity of CNTs reinforced gelatin-based hydrogels under stretching; twisting; and compression. Reprinted with permission from [171]. Copyright 2020 Elsevier. (**c**) Healing process of a cellulose-based hydrogel and (**d**) the tensile stress vs. strain behavior of original and self-healed hydrogels with different healing time, from 10 m to 60 m. Reprinted with permission from [172]. Copyright 2020 Royal Society of Chemistry.

Eco-friendly CHG are often designed to be highly flexible and stretchable, allowing them to conform to curved surfaces or deform with the skin’s movement without losing their functionality [52]. The natural polymer matrix and crosslinking methods are critical for achieving these properties. Hydrogels with high stretchability are used in soft, skin-like WE that need to bend and stretch without cracking, such as flexible electrodes, sensor patches, and electronic skins (e-skins) [169,170]. Toughness refers to the ability of the hydrogel to absorb energy without breaking, which is important for WE that need to endure mechanical stresses over time. Eco-friendly CHG can be engineered for high toughness by controlling the crosslinking density, polymer composition, and the type of conductive filler used [74]. In applications like wearable sensors or actuators, where hydrogels are subject to repeated stretching, bending, and twisting, high toughness ensures longevity and prevents mechanical failure. Figure 7 illustrates the stretchability of CNT-reinforced gelatin-based [171] and cellulose-based CHG with outstanding mechanical properties and rapid self-healing performance [62,163]. Carboxymethyl cellulose can interact with adjacent polymer networks and metal ions through hydrogen bonding and dynamic noncovalent interactions due to the rich hydroxyl and carboxyl groups on the backbone chains, which is advantageous in the design and synthesis of conductive hydrogels with both high mechanical and self-healing properties. Using carboxymethyl cellulose and Al^3+^ as cross-linkers, in addition to polyacrylic acid due to their abundant ligand carboxyl groups, a cross-linking network was formed between polymeric chains by hydrogen interactions, and a secondary crosslinking network was introduced by dual ionic coordination bonds between Al^3+^ and COO− from polymeric chains. In the carboxymethyl cellulose-Al^3+^-polyacrylic acid interwoven cross-linked networks, the strong ionic coordination bonds as a primary network and the weak hydrogen bonds as a sacrificial network, endowing the conductive hydrogel with fast self-recovery, and good self-healing performance [172].

### 4.3. Skin-Friendliness

Biocompatibility is essential for materials used in WE, particularly those that come in direct contact with the skin. Eco-friendly CHG made from natural polymers (e.g., alginate, chitosan, or gelatin), are inherently biocompatible and non-toxic. Furthermore, these hydrogels can be modified to be hypoallergenic, reducing the risk of skin irritation or adverse reactions when worn for extended periods. Eco-friendly CHG are used in wearable health-monitoring devices, such as biosensors or sweat analyzers, where skin contact is prolonged [174,175]. The materials must not cause discomfort or irritation, ensuring user safety. Since eco-friendly CHG are often designed to be biodegradable, they can degrade safely in the environment or within the human body over time, making them ideal for disposable or medical applications. Non-toxic crosslinkers and biopolymers ensure that the materials are safe for short- or long-term use, reducing the environmental impact. In biomedical applications like wound healing or implantable sensors, biodegradable hydrogels offer the advantage of safe dissolution after use, eliminating the need for removal or disposal of electronic devices [58,176].

**Figure 8:**
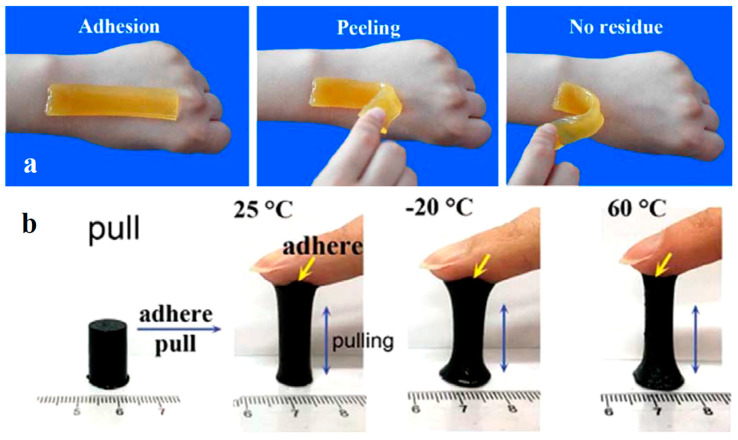
(**a**) Hydrogel showing tissue adhesiveness on the hand skin and no residue after removal. Reprinted with permission from [177]. Copyright 2021 Elsevier. (**b**) Glycerol-based hydrogel showing high tissue adhesiveness under temperatures from −20 to 60 °C. Reprinted with permission from [178]. Copyright 2018 Wiley.

Skin-like hydrogel devices still face challenges, including poor surface adhesion [169]. Zhang et al. prepared a stretchable, wearable hydrogel strain sensor with double layers via in situ polymerization of nucleobase-driven adhesive hydrogels on the surface of conductive tough hydrogels crosslinked by hydrophobic association [177]. The hydrogels displayed tissue adhesiveness on the hand skin and no residue was observed after removal (Figure 8a). Han et al. prepared a glycerol-based hydrogel based on mussel chemistry and incorporating polydopamine-decorated CNTs, endowed with high tissue adhesiveness under wide temperature spectrum (−20 or 60 °C) (Figure 8b). The mussel-inspired hydrogel is a promising material for self-adhesive bioelectronics in cold or hot environments, and can also serve as a dressing to protect the skin from injuries related to frostbite or burns [178].

Green hydrogels, derived from natural polymers, exhibit excellent biodegradability (the ability of the hydrogel to break down into non-toxic components through enzymatic, microbial, or hydrolytic processes) and biocompatibility, making them ideal for biomedical and environmental applications. These properties are significantly influenced by the type of natural polymer used, its crosslinking density, and the surrounding conditions. Natural hydrogels degrade in biological environments due to enzymes (e.g., lysozyme for chitosan, cellulase for cellulose), by hydrolytic degradation in aqueous conditions without enzymatic assistance (e.g., gelatin, alginate), or by microbial degradation: certain natural polymers (e.g., starch, cellulose) decompose in soil or water due to microbial activity [179]. The degradation rate (% weight loss over time) varies from gelatin-based hydrogels (50–90% weight loss in 1–4 weeks in vivo) or alginate hydrogels (~80% degradation in 1–3 weeks) to chitosan-based hydrogels (30–70% weight loss in 2–6 weeks, dependent on crosslinking) and cellulose-based hydrogels, showing slow degradation (~10–40% in several weeks) due to high crystallinity. Hydrophilic natural polymers (e.g., gelatin, alginate) undergo rapid degradation due to water absorption and hydrolysis, while structural natural polymers (e.g., cellulose, chitosan) show slower degradation due to higher crystallinity and resistance to microbial attack.

Therefore, degradation half-life can be estimated to range from approximately ~1 week for alginate to several months for cellulose. Environmental conditions affecting degradation include pH sensitivity, alginate and chitosan degrade faster in acidic conditions; enzyme presence: chitosan degrades more rapidly in lysozyme-rich environments, and crosslinking density: higher crosslinking reduces degradation speed (e.g., glutaraldehyde crosslinked hydrogels degrade slower).

Regarding biocompatibility, the ability of the hydrogel to interact with biological tissues without causing toxicity, inflammation, or immune rejection, some natural hydrogels (e.g., gelatin, collagen) promote cell attachment and exhibit excellent biocompatibility, with cell viability after 24 h above 90%, while some (e.g., alginate) require modifications for better cell interaction. Inert polymers (e.g., alginate, cellulose), highly biocompatible but showing low cell adhesion, are often modified with RGD peptides for better tissue interaction. Chitosan-based hydrogels can trigger mild immune activation and may need neutralization for reduced cytotoxicity and inflammatory response via deacetylation. On the other hand, chitosan has antibacterial properties [180].

### 4.4. Swelling Behavior and Stimuli Responsive Properties

Hydrogels are known for their ability to retain large amounts of water within their structure, providing flexibility, stretchability, and softness. This high water content is important for maintaining the desired mechanical properties and ensuring that the hydrogels remain functional in humid environments or during physical activities. In WE, maintaining hydration is crucial for both comfort and device performance. For bioelectronics like skin sensors, high water content is essential for ensuring that the hydrogel can maintain conductivity even in the presence of sweat or moisture from the skin. The ability of CHG to swell or change their volume upon exposure to moisture or environmental changes is another important functional property. The swelling behavior of the hydrogel influences its mechanical stability, conductivity, and ability to interface with the body or skin. Swelling behavior is critical in applications like wound dressing [173,177,178] or sweat sensing devices [181,182], where the hydrogel must respond to changing moisture levels. Hydrogels with controlled swelling can also be used in drug delivery systems, where they release bioactive compounds in response to the swelling process. CHG for WE must retain their stability over a wide range of environmental conditions, including varying temperature and humidity. Eco-friendly CHG are often engineered to be stable under these conditions by incorporating additives or optimizing the network structure. This stability ensures reliable performance in real-world applications where users may experience sweating, temperature fluctuations, or prolonged wear. In wearable devices such as smart patches or biosensors, stability ensures that the hydrogel maintains its functionality over time, even during prolonged use or exposure to varying environmental factors like moisture or temperature.

Some CHG are designed to be sensitive to changes in pH or ionic concentration or designed to change shape or size in response to an external stimulus, such as electric fields, temperature changes, or humidity [183,184,185,186,187]. These hydrogels respond to physiological signals or environmental changes, making them useful for applications in sensors, actuators, or bioelectronics. Hydrogels that respond to changes in pH or ionic strength are used in wearable sweat sensors that detect biomarkers in sweat or skin sensors that monitor physiological conditions, such as dehydration or glucose levels. These responsive hydrogels can serve in WE as actuators in applications like haptic feedback devices, where the hydrogel changes its shape to provide tactile sensations to the user.

Incorporating natural ingredients into hydrogels significantly influences their swelling behavior and stimuli-responsive properties. This correlation is largely due to the intrinsic characteristics of natural polymers, such as hydrophilicity, functional groups, porosity, and crosslinking density. Natural polymers (e.g., alginate, chitosan, gelatin) have abundant hydroxyl (-OH), carboxyl (-COOH), and amine (-NH_2_) groups, which enhance water absorption and retention. Many green hydrogels have a porous structure, allowing for higher water uptake. A higher crosslinking density (e.g., ionic crosslinking in alginate with Ca^2+^) restricts swelling, while loosely crosslinked networks (e.g., physically crosslinked gelatin) enhance swelling. Several examples of swelling enhancement by natural polymers include alginate hydrogels, where swelling is highly dependent on ion concentration and decreases in the presence of calcium ions; chitosan hydrogels, which are pH-sensitive and swell more in acidic conditions due to the protonation of amine groups; and cellulose-based hydrogels, which show high swelling due to abundant hydroxyl groups but can be modified for controlled water retention [181,188].

Natural ingredients also influence hydrogel responsiveness. Many natural polymers have ionizable groups that respond to pH changes. Hydrogels like alginate shrink or swell depending on ionic concentration due to crosslinking density changes [181]. Some natural polymers are thermo-responsive and/or pH-responsive: gelatin hydrogels undergo a transition from sol (liquid) to gel (solid) at physiological temperatures [189]. Methylcellulose hydrogels decrease swelling at high temperatures due to hydrophobic interactions. Chitosan-based hydrogels swell in acidic environments due to the protonation of amine (-NH_2_) groups [190]. Alginate-based hydrogels reduce swelling at low pH due to the formation of insoluble alginic acid. Generally, incorporating natural ingredients enhances hydrophilicity, swelling, and stimuli responsiveness, making green hydrogels more adaptable for biomedical applications (drug delivery, wound healing) and environmental applications (adsorbents, water purification). However, fine-tuning crosslinking and composition is essential to achieve the desired balance between swelling and mechanical stability.

## 5. Applications in WE for Healthcare

**Figure 9:**
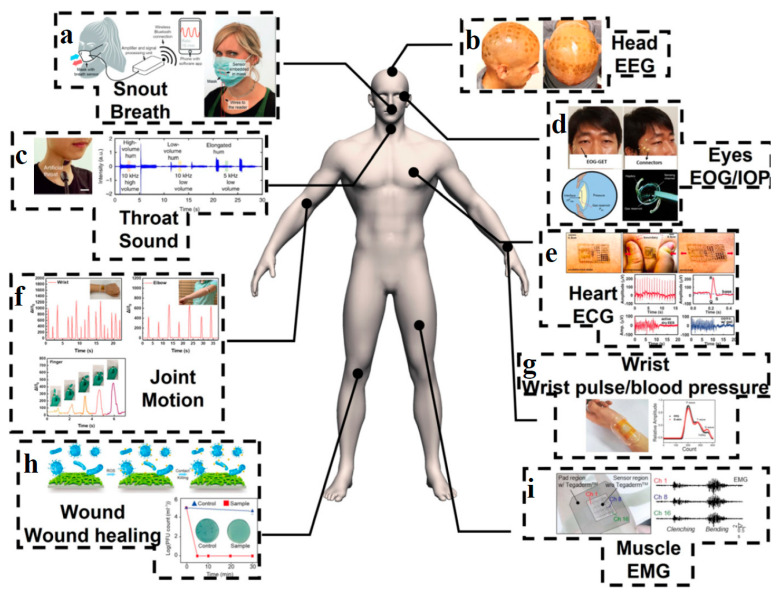
WE monitoring devices for (**a**) breath, reprinted with permission from [192], copyright 2016 Wiley; (**b**) EEG, reprinted with permission from [193], copyright 2019 Springer Nature; (**c**) sound, reprinted with permission from [194], copyright 2017 Springer Nature; (**d**) EOG/IOP, reprinted with permission from [195], copyright 2014 Springer Nature; (**e**) ECG, reprinted with permission from [196], copyright 2011 American Association for the Advancement of Science; (**f**) joint motion, reprinted with permission from [197], copyright 2021 Royal Society of Chemistry; (**g**) wrist pulse and blood pressure, reprinted with permission from [198], copyright 2014 Wiley; (**h**) wound healing monitoring, reprinted with permission from [199], copyright 2018 American Association for the Advancement of Science; (**i**) EMG, reprinted with permission from [200], copyright 2021 American Association for the Advancement of Science. Reprinted with permission from [191].

Eco-friendly CHG are increasingly being used in WE due to their unique combination of properties such as flexibility, biocompatibility, conductivity, and environmental sustainability. These properties make them ideal for creating sensitive, comfortable, and sustainable sensors that can monitor a variety of physiological and environmental parameters [58,70]. Figure 9 [191] illustrates various wearable systems for human health monitoring, including blood pressure breath, sound, wrist pulse, electroencephalogram (EEG), electrocardiograph (ECG), electromyography (EMG), electrooculogram / intraocular pressure (EOG/IOP), and wound healing.

Some key applications as biosensors for monitoring electrophysiological signals, skin biomarkers, pressure changes, minute movements, temperature, pH, moisture, UV radiation, or exposure to toxins include:

### 5.1. ECG Sensors

**Figure 10:**
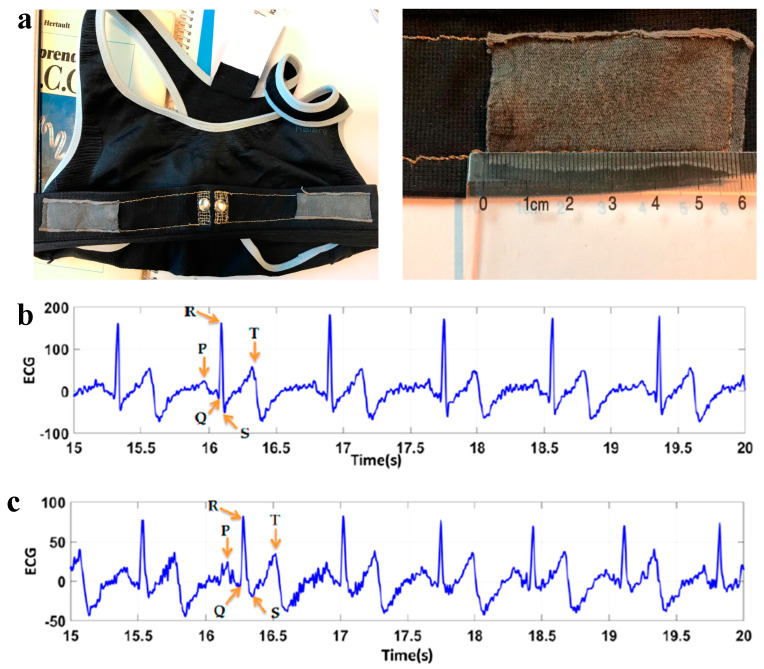
(**a**) PEDOT:PSS-modified cotton electrodes sewn into bras, reprinted with permission from [16]. ECG signal collected from PEDOT:PSS-coated textile electrode (**b**) before and (**c**) after 50 washing cycles, reprinted with permission from [202].

CHG are used to create skin-friendly ECG sensors that monitor heart activity [201]. Their ability to maintain good skin contact and conduct electrical signals ensures accurate readings. Ankhili et al. [16] reported the fabrication of lightweight, flexible, stretchable, conformable, washable, and long-lasting wearable electrodes using conducting PEDOT:PSS gels on commercial knitted fabrics, allowing a good contact with the skin (Figure 10). Washability tests of connected underwear were carried out up to 50 washing cycles, and ECG data, recorded from a healthy volunteer, were found to be stable [202]. Textile fabrics coated with PEDOT:PSS gels were assembled into bras textiles structures, then textiles were connected to measurement devices and high-quality ECG signals were recorded [203]. Simple and reliable techniques allow the patterning of conducting polymers on textiles and the fabrication of electrodes that provide a low-impedance contact with human skin, enabling the recording of high-quality ECGs [204]. Three-dimensional printable hydrogels based on PEDOT:PSS have shown superior 3D printability for direct ink writing [205,206].

### 5.2. EEG Sensors

**Figure 11:**
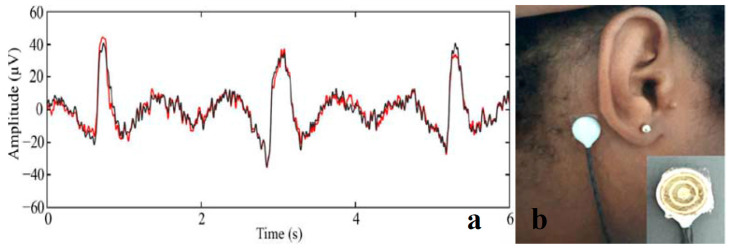
(**a**) EEG containing eye blinks recorded using an alginate hydrogel, reprinted with permission from [207]. Copyright 2017 Elsevier. (**b**) The inset in the lower right shows the hydrogel tape on the sensor placed on the right mastoid process for EEG recording, reprinted with permission from [208].

For brain activity monitoring, natural hydrogel-based EEG sensors offer a non-irritating and reusable alternative to traditional gel-based or Ag/AgCl wet electrodes. An alginate-based hydrogel developed for EEG applications ensures a faster and easier cleaning than commercial gels, eliminating the head washing and drying process, improving patient comfort. Additionally, the solid nature of the alginate hydrogel reduces the risks of electrode short-circuits, thus enhancing EEG reliability [207]. Hydrogel tapes offer a promising alternative to conductive paste, providing mess-free application and reliable electrode–skin contact in locations without hair (Figure 11) [208].

### 5.3. Electromyogram (EMG) Sensors

**Figure 12:**
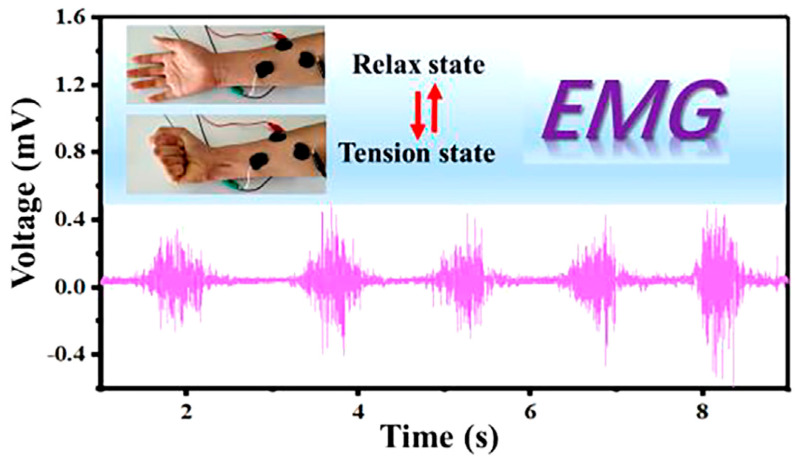
Hydrogel electrodes used to detect signals for the EMG, reprinted with permission from [210]. Copyright 2020 Elsevier.

These sensors detect muscle activity, which is useful in physical therapy and sports training. The flexibility of hydrogels allows them to conform to the body’s movements without compromising signal quality. Surface electromyography (EMG) has not yet been effectively implemented in practical medical settings [209]. Pan et al. [210] reported a wearable and strain-sensitive hydrogel-based electronic skin which can mimic and detect some real skin epidermis movements such as finger bending, facial expression changes, and throat vocalization. The hydrogel can also be used as an adhesive electrode for the accurate detection of ECG and EMG signals (Figure 12).

### 5.4. Glucose Monitoring

**Figure 13:**
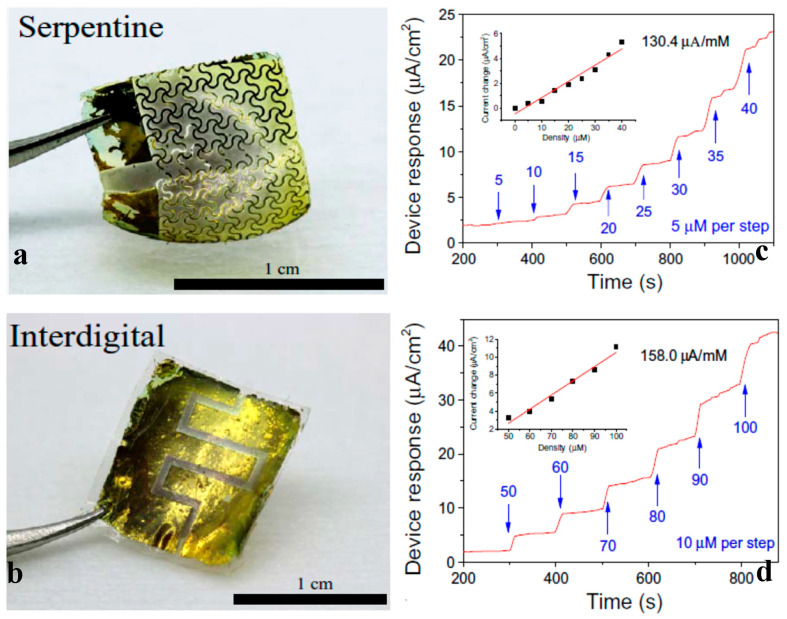
Chitosan–glucose oxidase hydrogel biosensors with serpentine (**a**) and interdigital (**b**) pattern and calibration amperometric I vs. t results of (**c**) low-density and (**d**) moderate-density glucose. (**Inset**) Biosensor response as a function of density. Reprinted with permission from [212]. Copyright 2017 American Association for the Advancement of Science.

CHG are integrated into wearable glucose monitors that measure glucose levels through sweat or interstitial fluid, offering a non-invasive alternative to traditional blood tests [211]. Chen et al. [212] presented a skin-like biosensor system (Figure 13), based on chitosan-containing glucose oxidase, for non-invasive and highly accurate intravascular blood glucose monitoring for continuous clinical-grade use. Kim et al. [213] reported smart contact lenses with long-term continuous glucose monitoring using nanoporous hydrogels.

### 5.5. Sweat Analysis

These hydrogels can detect various biomarkers in sweat, such as electrolytes, metabolites, and pH levels, which are important for monitoring hydration, stress, and metabolic health [214]. Wearable patch-based sensors have emerged as a promising solution for effective sweat management, offering easy induction, reliable collection, and precise analysis [215]. Hydrogel patches can simultaneously serve as an interface for sweat sampling and a medium for electrochemical sensing [216]. Silk fibroin-based double network hydrogel adhesive with strong and durable adhesion on wet surfaces can be integrated with epidermal sensor arrays and perform in real-time on-body sweat sensing [217]. PEDOT-based wearable hydrogel patches were used in noninvasive, electrochemical glucose sensors for natural sweat detection by enabling the analysis of sweat glucose during routine and sedentary activities [218].

Lactic acid sensors. Used by athletes, these sensors help monitor muscle fatigue by detecting lactic acid levels in sweat, providing real-time feedback during training [219]. Saha et al. developed a lactate monitoring platform that collects sweat over extended periods using hydrogels for osmotic extraction and paper microfluidic channels for sample evaporation to address insufficient sweating [220].

### 5.6. Soft Pressure and Motion Sensors

**Figure 14:**
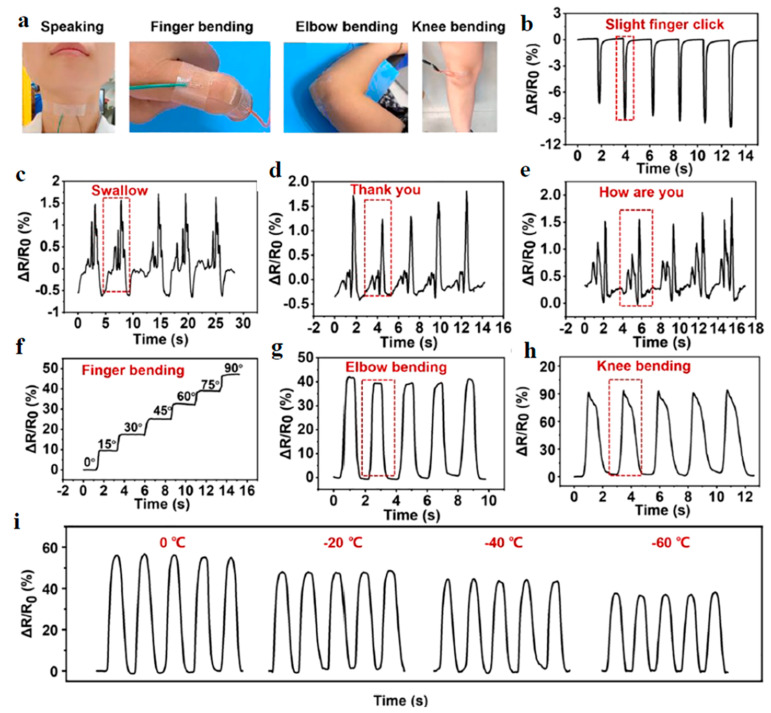
(**a**) Resistive sensors to monitor human motions. (**b**–**i**) Resistance changes by (**b**) slight finger click; (**c**) swallowing; speaking the words (**d**) ‘thank you’, and (**e**) ‘how are you’; (**f**) finger, (**g**) elbow, (**h**) knee bending, and (**i**) finger bending at different subzero temperatures, reprinted with permission from [161]. Copyright 2021 Elsevier.

**Figure 15:**
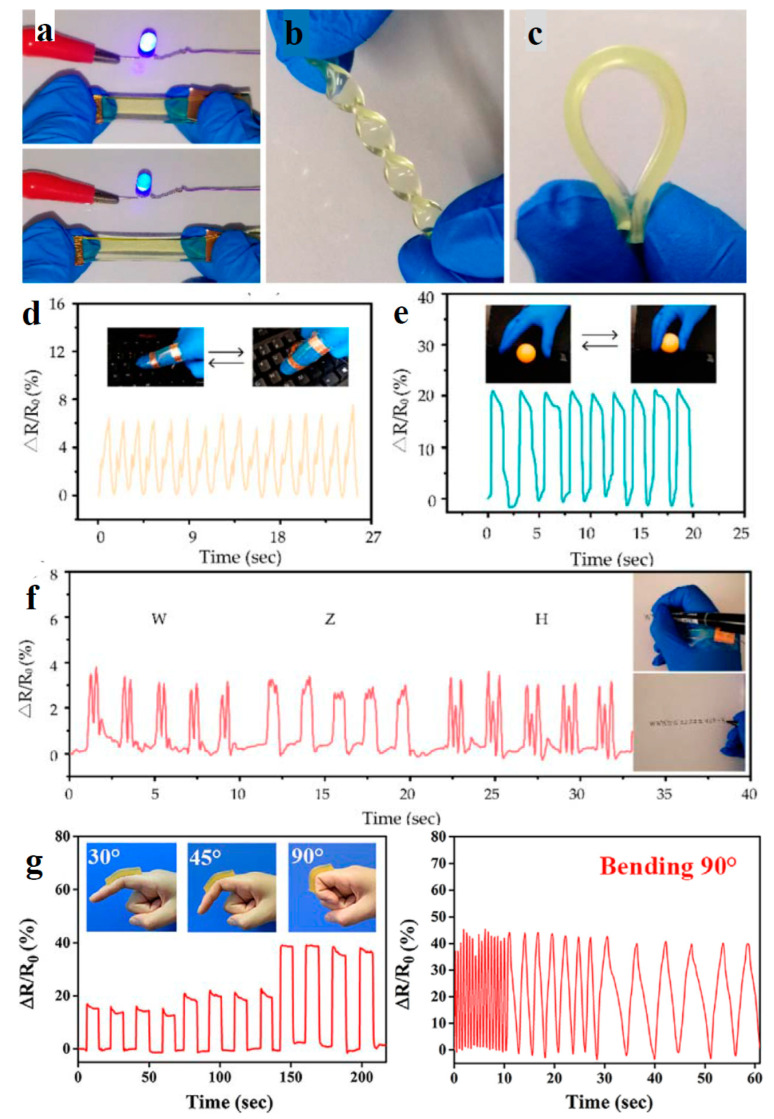
Cellulose-based hydrogels: (**a**) ionic-conductive and stretchable, (**b**) twistable, and (**c**) flexional. Resistance variations in the strain sensor: (**d**) tapping the keyboard, (**e**) grasping, and (**f**) writing the letters “w”, “z”, and “h” [81]. (**g**) Bending the finger at different angles and 90° bending speeds. Reprinted with permission from [180]. Copyright 2019 Elsevier.

CHG can be used in sensors that monitor pressure changes, making them useful in detecting body posture, gait analysis, or even subtle changes in breathing patterns. *Motion sensors* to detect bending, stretching, and twisting are useful in sports and rehabilitation devices (Figure 14 and Figure 15).

Artificial skin-like materials have wide applications, particularly in flexible electronics. However, developing intelligent skin-like soft materials with a remarkable range of properties is still a challenge. Lin et al. [82] prepared a biomimetic skin-like hydrogel based on Ag/TA@cellulose nanocrystals, decorated with tannic acid (TA) and Ag nanoparticles nanohybrids, combined with PVA via chemical crosslinking, which achieved the combination of superstretchability (>4000%), efficient (within 10 min, 98.6%), and repeatable self-healing property, conformability, and the ability to sense and track human body motions with a relatively broad range of strain (up to 400%). The hydrogel can also be used for repairing circuits, constructing switches, programmed electrical circuit assembly, as electronic skin, and in touch screen pens. A Ti_3_C_2_T*_x_* MXene-based PEDOT:PSS composite conductive aerogel was prepared using Cu-assisted electrogelation and assembled into pressure sensors for high-resolution robotic tactile sensing to directly recognize the tactile stimuli from human fingers and identify braille letters like human fingers [221].

**Figure 16:**
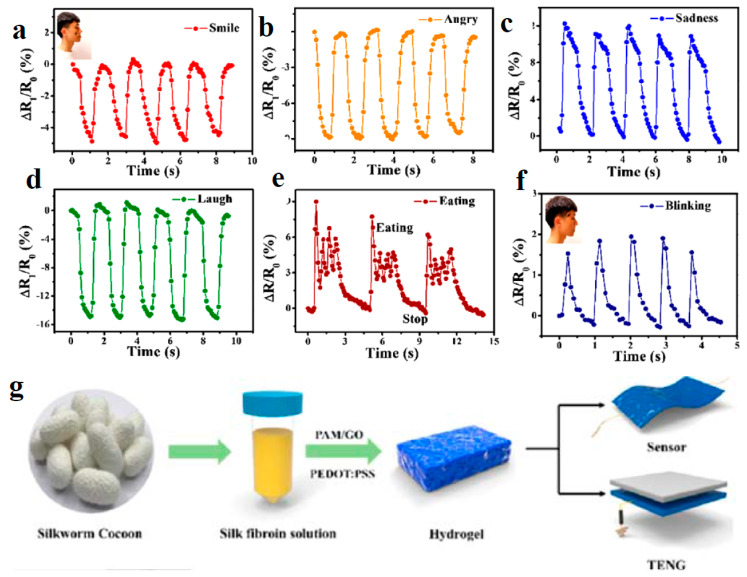
Application of a silk fibroin-based hydrogel incorporating PEDOT:PSS and graphene oxide (GO) sensor for monitoring body signals. (**a**–**f**) Relative resistance variations for different facial gestures: smile, anger, sadness, laugh, eating, and blinking. (**g**) Schematic illustration of the fabrication of the silk fibroin-based hydrogel, sensor, and TENG. Reprinted with permission from [91]. Copyright 2020 American Chemical Society.

Strain sensors can detect minute deformations caused by body movements (Figure 16), making them ideal for monitoring joint movements or detecting early signs of physical stress or strain [222,223,224].

### 5.7. Temperature and Thermal Therapy Monitoring

**Figure 17:**
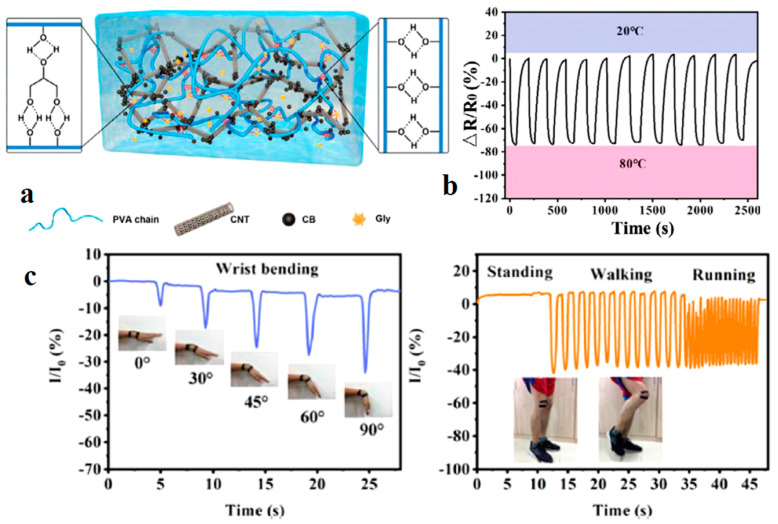
(**a**) Scheme of the CHG: PVA, glycerol-based hydrogel incorporating carbon black, and CNTs. (**b**) Dynamic thermal response test of the CHG between 20 °C and 80 °C. (**c**) Current vs. time response of the CHG-based strain sensor in: wrist bending, and knee movements, including standing, walking, and running. Reprinted with permission from [225]. Copyright 2020 American Chemical Society.

Wearable devices with hydrogel-based temperature sensors can track body temperature continuously, which is useful in healthcare monitoring for fever or hypothermia. For applications in thermal therapy, hydrogels can help ensure that the target area maintains the desired temperature range. The incorporation of a flexible temperature sensor is a significant breakthrough for realizing intelligent wearable devices. An environmental-friendly CHG was prepared by freeze–thaw, introducing CNTs and carbon black into a poly(vinyl alcohol)/glycerol hydrogel [225] for wearable strain and temperature sensors. Glycerol incorporation enables long-lasting moisture retention and low temperature tolerance. The CHG exhibited a linear relationship between temperature and relative resistance change during both the heating and cooling processes, demonstrating a constant temperature coefficient of resistance (TCR) of 0.945(10) %°C^−1^. A dynamic thermal response test between 20 °C and 80 °C (Figure 17), showed a clear signal response, demonstrating an excellent temperature discrimination capacity.

### 5.8. Smart Bandages

**Figure 18:**
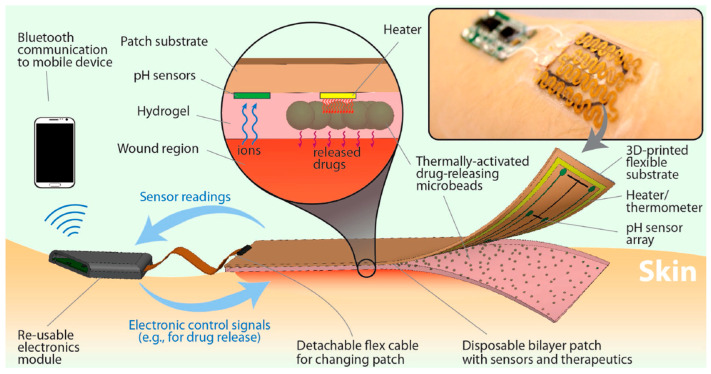
Conceptual schematic of a smart bandage. Flexible pH sensors and a heater trigger the delivery of antibiotics from thermo-responsive carriers embedded in a layer of alginate hydrogel. An automated electronic module records the sensor signals, powers the heater when needed, and is able to communicate wirelessly. Reprinted with permission from [227]. Copyright 2018 Wiley.

CHG are incorporated into smart bandages that monitor the wound healing process by detecting changes in wound environment, such as pH (pH changes in a chronic wound are typically interpreted as an indication of bacterial infection), temperature, and moisture levels. The data help in adjusting treatment protocols accordingly. Smart bandages able to interact with the wound automatically is an innovative concept for effective wound care and accelerated wound healing [226]. A smart bandage was engineered with multiple components, including sensors (pH and temperature), microheater, thermo-responsive drug carriers embedded in a hydrogel patch, and wireless electronics to read the data from the sensors and to trigger and control the thermal actuation system if required (Figure 18) [227].

### 5.9. Electronic Skin

**Figure 19:**
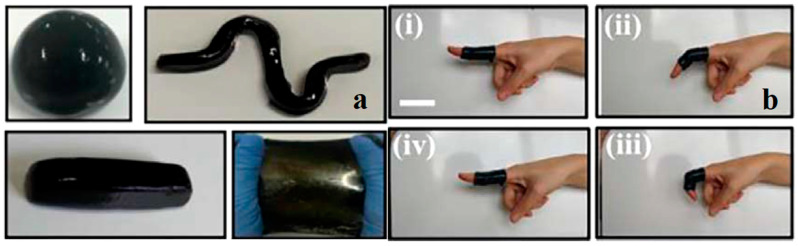
(**a**) Proanthocyanins/rGO/PVA hydrogel adjusted to sphere, cuboid, and curve shapes and stretched into a skin-like film. (**b**) The hydrogel dynamically adapts to the 3D surface of the finger and simultaneously moves with it: (**i**) pristine state, (**ii**) 45° bending, (**iii**) 90° bending, and (**iv**) pristine state (scale bar: 5 cm). Reprinted with permission from [210]. Copyright 2020 Elsevier.

Du et al. [228] reported wearable TENGs based electronic skin (e-skin) patches with PPy/Pluronic F127 hydrogel for accelerating wound healing by locally generating electrical fields to the wound area. These electronic skin patches used self-power electric generation and photothermal wound healing acceleration within 11 days. A bionic tactile Proanthocyanins/rGO/PVA hydrogel-based electronic skin (Figure 19), which simulates the tactual sensation of human skin and integrates stretchability (>5000%) and a self-healing (3 s, 95.73%) ability, can mimic and detect real skin epidermis movements such as finger bending, facial expression changes, and throat vocalization. Interestingly, the hydrogel can also be used as an adhesive electrode for the accurate detection of ECG and EMG signals [210].

However, the development of skin-like hydrogel devices is still in its infancy and faces challenges including limited functionality, low ambient stability, poor surface adhesion, and relatively high power consumption (as ionic sensors) [169].

### 5.10. UV and Pollution Sensors

Hydrogels can be engineered to respond to UV radiation or environmental pollutants, providing real-time data on exposure, alerting the wearer to dangerous conditions, and helping users take preventive measures. Apart from being used as wearable sensors, these sensors have the potential to be used along with UV-based workspace sterilizing devices to ensure that surfaces have been efficiently exposed to UV. UV sensors made by Finny et al. using alginate, gelatin, photoactive titanium dioxide nanoparticles, and dyes (methyl orange, methylene blue, and malachite green), in which the nanoparticles are used to initiate photocatalytic degradation of dyes, leading to discoloration of the dye, visible to the naked eye, are inexpensive, stable, extremely robust, biodegradable and easy to use [229].

### 5.11. Breath Sensors and Toxin Detection

**Figure 20:**
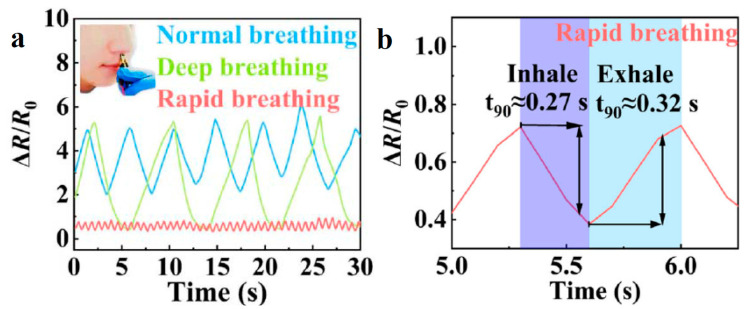
Humidity sensor (**a**) monitors and distinguishes breath status. (**b**) Response and recovery time during rapid breath monitoring. Reprinted with permission from [224]. Copyright 2018 Springer.

They can be integrated into wearable devices that monitor exposure to hazardous chemicals or toxins, alerting the wearer to potential health risks. Wearable vapor sensors can help people monitor air composition in real time to avoid underlying risks, and for the early detection and treatment of diseases for home healthcare [230]. A sensitive humidity responsive sensor based on sodium hyaluronate incorporating multi-walled CNTs composite hydrogel was deposited on a flexible interdigital electrode by a drop coating method [231]. The sensor presents a broader detection range for relative humidity (11–98% RH) and fast response/recovery performance (0.32 s/0.27 s), stability after repeated humidity changes, long-term operation, temperature changes, and cyclic mechanical bending (Figure 20).

**Figure 21:**
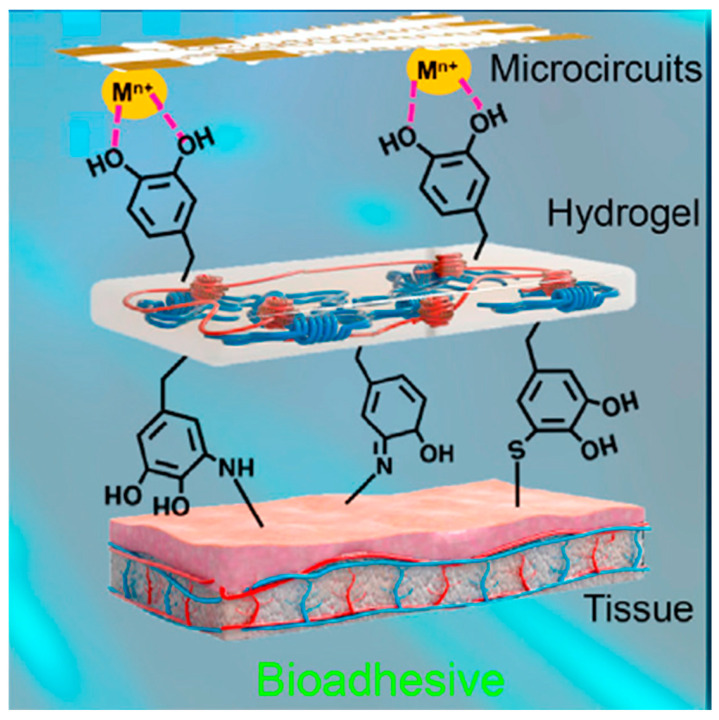
Adhesion mechanism of mussel inspired hydrogel to soft tissue and metal substrates. Reprinted with permission from [237]. Copyright 2022 Elsevier.

These applications demonstrate the versatility of eco-friendly CHG in wearable bioelectronic sensors, contributing to advanced health monitoring and environmental interaction while emphasizing sustainability and user comfort. Table 2 summarizes some recent biomedical applications of WE. We are enjoying rapid advances towards the goal of imperceptible WE for human health monitoring. Wearable and implantable bioelectronics have emerged as an alternative or adjunct to conventional healthcare. Bioelectronic systems must be designed eco-friendly for sustainable healthcare [232,233]. Furthermore, CHG can be used in the electrodes of flexible and stretchable displays, offering more comfortable and adaptable wearable screens. In wearable robotics, hydrogels can serve as actuators, mimicking natural muscle movement due to their ability to expand and contract in response to electrical stimuli. To assist patients with restricted mobility to control wheelchair freely, Wang et al. developed an eye-movement-controlled wheelchair prototype based on a flexible hydrogel biosensor made of conductive Hydroxypropyl cellulose/PVA hydrogel and flexible Polydimethylsiloxane substrate [234]. Bioinspired hydrogels hold promise for designing bioadhesive brain–machine interfaces with immune-evasive capability, actively preventing fibrous tissue encapsulation and neuroinflammation after implantation, and enabling communication between the brain and external machines. Mussel-inspired polydopamine hydrogels exhibit high adhesion, attributed to the presence of sufficient free catechol groups as well as mechanical and biochemical affinity for biological tissues, including brain tissue [235,236]. This enables seamless adhesion to brain tissue and integration with metal microcircuits (Figure 21).

Due to their biocompatibility, eco-friendly CHG are suitable for implantable devices that monitor or stimulate internal organs, like pacemakers or neural interfaces. Smart wearable patches can use hydrogels to control the release of drugs, responding to stimuli like body temperature or electrical signals. These applications highlight the versatility of eco-friendly CHG in advancing the field of WE, focusing on both user comfort and sustainability.

## 6. Challenges and Limitations

### 6.1. Trade-Offs Between Performance and Ecofriendliness

The development of eco-friendly CHG for WE presents several challenges, particularly in balancing high performance with sustainability. While efforts to replace synthetic and non-biodegradable components with natural and renewable materials are advancing, certain trade-offs between performance and ecofriendliness remain:

Electrical Conductivity vs. Biodegradability. A significant issue with the increasing prevalence of WE is the problem of electronic waste (e-waste). It is crucial to prioritize the development of electronics that utilize biodegradable and environmentally friendly materials and processes to mitigate the environmental impact. Currently, most wearables are made of metal nanowires, conducting polymers, carbon-based nanomaterials, and liquid metals, frequently combined with substrates like fabric or elastomers. Many highly conductive materials, such as metal nanoparticles, are not biodegradable and may pose environmental risks. Natural alternatives, such as bio-derived carbon materials and polymers, often exhibit lower conductivity or stability, limiting their efficiency in electronic applications [238].

Mechanical Strength vs. Sustainable Synthesis. Petroleum-based polymers provide excellent mechanical robustness and flexibility, but they are non-biodegradable. Biopolymer-based hydrogels (e.g., chitosan, cellulose, alginate) are more sustainable but often lack sufficient mechanical strength, requiring chemical crosslinking, which may introduce toxicity or reduce recyclability.

Stability and Longevity vs. Environmental Impact. Enhancing hydrogel durability is essential for long-term WE but often requires synthetic stabilizers or additives that hinder biodegradability. Sustainable alternatives, such as enzymatically crosslinked hydrogels, may degrade too quickly under certain conditions, limiting their practical use.

Scalability and Cost vs. Green Manufacturing. Large-scale production of eco-friendly hydrogels often requires expensive bio-sourced materials or complex synthesis routes that are less cost-effective compared to conventional methods. Green processing techniques, such as solvent-free fabrication or bio-based crosslinking, may be less efficient or require optimization to meet industrial demands.

Recyclability vs. Functional Properties. Designing CHG that are both recyclable and high-performing is a major challenge, as most existing hydrogels do not retain conductivity and mechanical properties after recycling. Developing self-healing and reprocessable hydrogels could address this issue, but current solutions often involve trade-offs in conductivity or structural integrity.

Balancing performance, durability, and sustainability remains a key challenge, but innovative material design and green chemistry approaches hold promise for next-generation eco-conscious WE.

To overcome these trade-offs, researchers are exploring hybrid materials that combine biodegradable polymers with sustainable conductive fillers and green synthesis strategies, such as enzymatic or supramolecular crosslinking, to enhance durability without compromising ecofriendliness and circular economy approaches, including recyclable and self-healing hydrogels, to extend material life cycles.

### 6.2. Scalability of Green Synthesis Methods

The transition toward eco-friendly CHG for WE relies heavily on sustainable synthesis approaches. However, scaling up these green synthesis methods from lab-scale research to industrial production presents several challenges:

- Many green synthesis methods depend on natural polymers (e.g., cellulose, chitosan, alginate) or bio-derived conductive materials (e.g., carbon nanodots, polydopamine). Ensuring a stable supply chain for biodegradable and renewable raw materials with consistent properties is critical for scalability. Variability in natural sources, extraction efficiency, and purity can lead to inconsistencies in hydrogel properties, affecting performance and reproducibility.
- Adapting and scaling low-energy, cost-effective fabrication techniques that maintain performance while remaining eco-friendly is an ongoing challenge. Some bio-based synthesis methods still rely on harsh conditions, high temperatures, or significant water consumption, which may offset their eco-friendly advantages. Developing low-energy, water-efficient, and waste-minimizing hydrogel production processes is crucial for large-scale implementation. Sustainable synthesis approaches, such as enzyme-assisted polymerization, solvent-free processing, or supramolecular self-assembly, are difficult to scale due to their complexity and cost (e.g., longer reaction times, precise pH conditions, temperature control). Bio-based crosslinking agents (e.g., genipin, citric acid) and natural dopants may be more expensive than synthetic alternatives, limiting commercial viability.
- Adapting green hydrogel processing to existing manufacturing infrastructure (e.g., 3D printing, roll-to-roll processing) requires further optimization, due to limited industrial compatibility of eco-friendly hydrogels. Traditional hydrogel fabrication methods, such as chemical crosslinking with toxic reagents (e.g., glutaraldehyde), are well-established for mass production. Green alternatives, such as photo-crosslinking, ionic crosslinking, or biodegradable linkers, often result in weaker mechanical properties or slower gelation rates.

### 6.3. Durability and Long-Term Stability

Eco-friendly CHG are often prone to moisture absorption, microbial degradation, or oxidative instability, reducing their long-term usability. Chemical stabilization without compromising biodegradability remains a challenge, particularly for large-scale storage and transportation. To overcome these challenges, researchers are exploring bioinspired synthesis strategies that mimic natural self-assembly processes, enzyme-catalyzed or microorganism-assisted polymerization for sustainable material production, integration with scalable fabrication techniques like 3D printing, extrusion-based processing, or inkjet printing for wearable applications, and recyclable and self-healing hydrogel formulations to extend product life cycles and reduce waste. Developing scalable, cost-effective, and environmentally responsible hydrogel synthesis methods is essential for the widespread adoption of green WE in the future.

Each of these challenges is deeply interconnected, making it difficult to optimize one property without negatively impacting another. The key to addressing these trade-offs lies in hybrid approaches, such as bio-inspired conductive networks, multi-scale material integration, and dynamic crosslinking strategies. Advancements in green chemistry, nanotechnology, and additive manufacturing could further bridge the gap between sustainability and high-performance CHGs, paving the way for truly eco-friendly wearable electronics.

## 7. Future Directions and Opportunities

As eco-friendly CHG gain traction in WE, future advancements will focus on improving functionality, sustainability, and scalability. Two key areas of development are:

### 7.1. Integration of Multi-Functional Materials

The seamless physical integration at the interface between hydrogel sensors and rigid electronic devices remains an unresolved challenge. This is primarily due to the water-rich environment, natural flexibility, and relatively lower Young’s modulus of CHG compared to other conventional hydrogels.

To enhance the performance and versatility of CHG, integrating multi-functional materials is crucial: hybrid conductive networks combining bio-based conductive materials (e.g., carbon nanodots, polydopamine) with metallic nanoparticles, MXenes, or conductive polymers to optimize electrical and mechanical properties; stimuli-responsive hydrogels developing materials that respond to temperature, pH, moisture, light, or magnetic fields, enabling advanced sensing, actuation, and self-healing properties; self-powered systems, embedding triboelectric, piezoelectric, or thermoelectric nanomaterials to enable energy harvesting and eliminate the need for external power sources, and finally incorporating natural antimicrobial and biocompatible agents (e.g., plant extracts or chitosan) to improve wearability in biomedical applications.

### 7.2. Advances in Recycling and Reusability and Potential for Circular Economies in WE

One of the biggest challenges in sustainable WE is enhancing recyclability and reusability while maintaining material integrity: self-healing hydrogels by developing dynamic covalent bonds (e.g., boronate esters, Schiff bases) or supramolecular interactions to allow reprocessing and reusability without compromising conductivity; designing water-resistant yet recyclable materials is crucial for long-term usability, as many hydrogels degrade in aqueous environments, especially in biofluids; creating ionic-conducting hydrogels or bio-based conductive inks that can be easily extracted (e.g., dissolved), reassembled, or repurposed, or implementing closed-loop manufacturing green synthesis routes that minimize waste generation while ensuring recovery of valuable materials. Moving towards a circular economy in WE involves reducing waste, extending product lifecycles, and designing materials for end-of-life recovery: developing biodegradable, naturally decomposable CHG that reduce e-waste and pollution; encouraging replaceable and upgradable hydrogel-based electronic components to extend device longevity. By modular and repairable designs. Green manufacturing and disposal strategies ensure a minimal environmental footprint. Using low-energy fabrication, non-toxic synthesis methods, and sustainable packaging. Finally, utilizing regenerative and renewable material sources, such as waste-derived biopolymers (e.g., from food waste, algae, or plant residues) for a truly sustainable material cycle.

In conclusion, the future of eco-friendly CHG holds great promise for sustainable, high-performance WE by integrating multi-functional materials, enhancing recyclability, and implementing circular economy principles. These advancements will drive the next generation of green, durable, and intelligent electronic devices while minimizing environmental impact.

## Figures and Tables

**Table 2:** Recent biomedical applications of WE.

Application	CHG	WE Description	Reference
ECG sensor	Polyamide, polyester, and cotton knitted fabrics coated with chemically modified PEDOT:PSS solution and silver-coated polyamide yarns used for signal transmission.	Low cost electrodes for cutaneous electrophysiology.	[16,202]
EEG sensor	Alginate-based hydrogels injected into the EEG electrode cavity.	Electrolytic gels for rapid EEG monitoring and easy cleaning procedures.	[205]
EMG and ECG sensor	Hydrogel-based electronic skin formed by proanthocyanins/reduced graphene oxide (PC/rGO) composite incorporated into glycerol-plasticized polyvinyl alcohol-borax (PVA-borax) hydrogel system.	Electronic skin, adhesive electrode.	[208]
Glucose monitoring	Gold platinum bimetallic nanocatalysts modified with hyaluronate immobilized in nanoporous hydrogels (HA-Au@Pt BiNCs).	Smart contact lenses for continuous tear glucose monitoring (CGM).	[215]
Sweat analysis	Silk fibroin-polyacrylamide (SF-PAAm) double network (DN) hydrogel adhesive.	Hydrogel patch-based sensor for real-time sweat detection on the body, biocompatible, with strong and durable adhesion to wet surfaces.	[215]
Soft pressure and motion sensor	Biomimetic hydrogel based on Ag/TA@CNC (cellulose nanocrystals (CNCs) decorated with tannic acid (TA) and Ag nanoparticles) nanohybrids and polyvinyl alcohol.	Soft artificial and intelligent material, similar to skin.	[82]
Temperature and tension sensor	Carbon nanotubes (CNT) and carbon black (CB) integrated into a poly(vinyl alcohol)/glycerol (PVA/Gly) nanocomposite organohydrogel.	Sensors with high sensitivity to stretch, strain and temperature.	[225]
UV and pollution sensor	3D printed tattoo-type sensors, based on hydrogel ink containing alginate nanoparticles, gelatin, photoactive titanium dioxide and dyes (methyl orange, methylene blue and malachite green).	Measures sun exposure by decreasing the color of the printed material.	[229]
Breath sensors and toxin detection	Sodium hyaluronate (SH)/multi-walled carbon nanotubes (MWCNTs) composite film.	Flexible interdigital electrode for non-contact monitoring of respiration and detection of skin sweat evaporation.	[231]

## Data Availability

No new data were created or analyzed in this study.

## Abbreviations

The following abbreviations are used in this manuscript:

CHG	Conductive Hydrogels
WE	Wearable Electronics
CNTs	Carbon Nanotubes
ECG	Electrocardiogram
EEG	Electroencefalogram
PVA	Polyvinyl Alcohol
PPy	Polypyrrole
PANI	Polyaniline
PEDOT:PSS	Poly(3,4-ethylene dioxythiophene):poly(styrene sulfonate)
EMG	Electromyogram
TENGs	Triboelectric Nanogenerators
EOG/IOP	Electrooculogram/Intraocular Pressure
